# The safety and efficacy of balloon-expandable versus self-expanding trans-catheter aortic valve replacement in high-risk patients with severe symptomatic aortic stenosis

**DOI:** 10.3389/fcvm.2023.1130354

**Published:** 2023-05-25

**Authors:** Nagendra Boopathy Senguttuvan, Hemal Bhatt, Vinod Kumar Balakrishnan, Parasuram Krishnamoorthy, Sunny Goel, Pothireddy M. K. Reddy, Vinodhini Subramanian, Bimmer E. Claessen, Ashish Kumar, Monil Majmundar, Richard Ro, Stamatios Lerakis, Ramamoorthi Jayaraj, Ankur Kalra, Marcus Flather, George Dangas

**Affiliations:** ^1^ Department of Cardiology, Sri Ramachandra Institute of Higher Education and Research, Chennai, India; ^2^ Department of Cardiology, The Zena and Michael A. Wiener Cardiovascular Institute, Icahn School of Medicine at Mount Sinai, New York, NY, United States; ^3^ Department of Cardiology, Hackensack Meridian Health, New Jersey, NJ, United States; ^4^ Department of Cardiology, Amsterdam University Medical Centres, Amsterdam, the Netherlands; ^5^ Department of Internal Medicine, Cleveland Clinic Akron General, Akron, OH, United States; ^6^ Department of Internal Medicine, New York Medical College, Metropolitan Hospital, New York, NY, United States; ^7^ Jindal Institute of Behavioral Sciences (JIBS), Jindal Global Institution of Eminence Deemed to Be University, Sonipat, India; ^8^ Department of Cardiovascular Medicine, Franciscan Health, Indiana, IN, USA; Co-CEO, Kalra Hospitals, New Delhi, India; ^9^ Professor of Cardiology, Norwich Medical School, University of East Anglia, Norwich, United Kingdom

**Keywords:** aortic stenosis, valve, balloon expandable, trans catheter aortic valve replacement, self-expanding

## Abstract

**Aim:**

Transfemoral Trans-catheter Aortic Valve Replacement (TF-TAVR) is a safe and effective therapy compared with surgical aortic valve replacement (SAVR) in patients across all risk profiles using balloon-expandable valves (BEV) and self-expanding valves (SEV). Our aim was to compare safety and efficacy of BEV vs. SEV in high-risk patients undergoing TF-TAVR.

**Methods and results:**

We searched PubMed, EMBASE, Clinicaltrials.gov, Scopus, and Web of sciences for studies on patients with severe aortic stenosis undergoing TAVR. Primary outcome was 30-day all-cause mortality. Secondary outcomes defined by Valve Academic Research Consortium 2 (VARC-2) criteria were also examined. Six studies with 2,935 patients (1,439 to BEV and 1,496 to SEV) were included. BEV was associated with lower risk of all-cause mortality (2.2% vs. 4.5%; RR: 0.51; 95% CI: 0.31–0.82; *p* < 0.006) and cardiovascular mortality [(2.5% vs. 4.3%; RR: 0.54; 95% CI: 0.32–0.90; *p* = 0.01) at 30 days compared with SEV. Implantation of more than one valve per procedure (0.78% vs. 5.11%; RR: 0.15; 95% CI: 0.07–0.31; *p* < 0.00001), and moderate/severe AR/PVL (2.5% vs. 9.01%; RR: 0.3; 95% CI: 0.17–0.48); *p* < 0.00001) were also lower in the BEV arm.

**Conclusion:**

BEV TAVR is associated with reduced all-cause mortality (High level of GRADE evidence), cardiovascular mortality (very low level) at 30 days compared with SEV TAVR in high surgical risk patients. Data are necessary to determine if the difference in outcomes persists in longer-term and if the same effects are seen in lower-risk patients.

**Systematic Review Registration:**

identifier, CRD42020181190.

## 1. Introduction

Trans-catheter aortic valve replacement (TAVR) is an established therapy for patients with symptomatic severe aortic stenosis (AS) across all surgical risk profiles (1). Three different platforms of trans-catheter heart valves are currently available: balloon-expandable valve (BEV), self-expanding valve (SEV) and mechanically expandable valve (MEV) (2). The United States Food and Drug Administration (FDA) has approved BEV devices including Sapien, Sapien-XT, Sapien-3 and Sapien-3 Ultra (Edwards Lifesciences, Irvine, CA, USA), SEV devices including CoreValve, Evolut R, Evolut Pro and Evolut-Pro+ (Medtronic, Minneapolis, MN, USA) in all AS patients, and LOTUS Edge™ (Boston Scientific, Boston, MA, USA) in high or greater risk patients (3–5). Other commonly used self-expanding devices outside the United States include Conformitè Europëenne (CE) marked devices like Acurate-Neo (Boston Scientific, Boston, MA, USA), Portico (Abbott Structural Heart, Santa Clara, CA, USA), Jena Valve (Jena Valve Technologies, Irvine, CA, USA), and Allegra (New Valve Technologies, Germany), and China FDA approved Venus-A (Venus Meditech, China) (2). Recently, Lotus Edge has been retrieved from the market. TAVR has overcome SAVR in the United States. But studies comparing the outcomes of different transcatheter valve systems are limited. It is well known that BEV is associated with few pacemaker requirements than SEV. But data regarding other hard-end points are scare. We therefore performed a systematic review and meta-analysis of randomized studies to study the safety and efficacy of TAVR using BEV vs. SEV devices in high risk patients.

## 2. Methods

### 2.1. Study eligibility

Studies were included, if they fulfilled the following criteria.

a) Randomized controlled trials (RCTs) in patients with severe native AS undergoing TAVR.
b) RCTs or *post hoc* analysis of RCTs comparing valve platforms into BEV vs. SEV or an RCT with pre-specified analysis by valve platforms. If a trial included MEV platform in either study arm (SEV or BEV), then it had to be <5% for inclusion in the current study.
c) Study should report all-cause mortality at 30 days as either primary or secondary outcome.

### 2.2. Search strategy

We searched PubMed, EMBASE, Clinicaltrials.gov, Scopus, and Web of science for all studies on patients with severe aortic stenosis undergoing TAVR (since inception to April 17th 2020) without any language restriction. We used multiple posting suffix (.mp) to improve sensitivity of our search. In addition, we looked for cross-references in the screened studies, review articles, and meta-analyses to identify other potential studies to be included. Our detailed search strategy is provided in the Supplementary Material. The study protocol is registered with PROSPERO, International prospective register of systematic reviews (CRD42020181190).

### 2.3. Eligibility assessment, data extraction and validity assessment

The Preferred Reporting Items for Systematic Reviews and Meta-Analyses (PRISMA) statement was followed during the development of this systematic review and meta-analysis (6). After eliminating duplicates, screening of manuscripts was done based on title and abstracts to remove irrelevant articles by two independent authors. Full text assessment of relevant, identified articles were scrutinized again by the above authors. Risk of bias assessment was done using Cochrane risk-of-bias tool for randomized trials version 2 (RoB 2) (7). Assessment of risk of bias, inconsistency, indirectness, imprecision, publication bias and effect size were assessed to calculate “certainty of evidence” using the GRADE (Grading of Recommendations, Assessment, Development and Evaluations) approach (8). Furthermore, the GRADEpro guideline development tool was used to create a “Summary of findings” table and a GRADE “Evidence profile”. Screening, full-text assessment, data extraction and validity assessment were independently performed by two authors (NBS and HB). Discrepancy was resolved by the third author (BC). We extracted baseline characteristics of patients, procedural details and clinical outcomes from included studies.

### 2.4. Outcomes

The primary outcome of our study was all-cause mortality at 30 days. Several endpoints on early safety, clinical efficacy and device success as defined by Valve Academic Research Consortium-2 (VARC-2) criteria were examined as secondary outcomes (9), including cardiovascular (CV) mortality, all stroke (disabling and non-disabling), life-threatening bleeding, major vascular complications, major bleeding, acute kidney injury (AKI) Stage 2 or 3 (including renal replacement therapy), myocardial infarction (MI), coronary artery obstruction requiring intervention, valve-related dysfunction requiring repeat procedure, moderate to severe aortic regurgitation (AR)/para-valvular leak (PVL), atrial fibrillation, rehospitalizations for valve-related symptoms or worsening congestive heart failure, permanent pacemaker implantation, prosthetic valve endocarditis, valve thrombosis, NYHA class III or IV, early valve-related dysfunction, implantation of more than one valve per procedure, valve malposition, and annular rupture. Whenever outcomes reported were too few in numbers (<5 events) or reported by a single study only, they were not included in the final analysis.

### 2.5. Statistical analysis

Data extracted from the studies were imported into Review Manager Version 5.3 (The Nordic Cochrane Center, The Cochrane Collaboration Copenhagen, Denmark) for analysis. Pairwise meta-analysis was performed for overall analysis. We used DerSimonian and Laird random effects model for our analysis to calculate pooled risk ratio (RR) and 95% confidence interval (CI) for all outcomes. We calculated between-study heterogeneity by using the Higgins I2 statistic. We defined low and high heterogeneity as *I*^2^ < 25% and >75% respectively. Publication bias was assessed visually by asymmetry in funnel plots. We performed sensitivity analyses utilizing various factors whenever statistically significant heterogeneity was found. This included an analysis after excluding studies one-by one that were considered an outlier based on methodological or interventional heterogeneity. A leave-one-out sensitivity analysis to remove the effect of one study at a time on our results was also performed. All tests were 2-tailed with a *p* value of <0.05 considered significant.

## 3. Results

**Figure 1:**
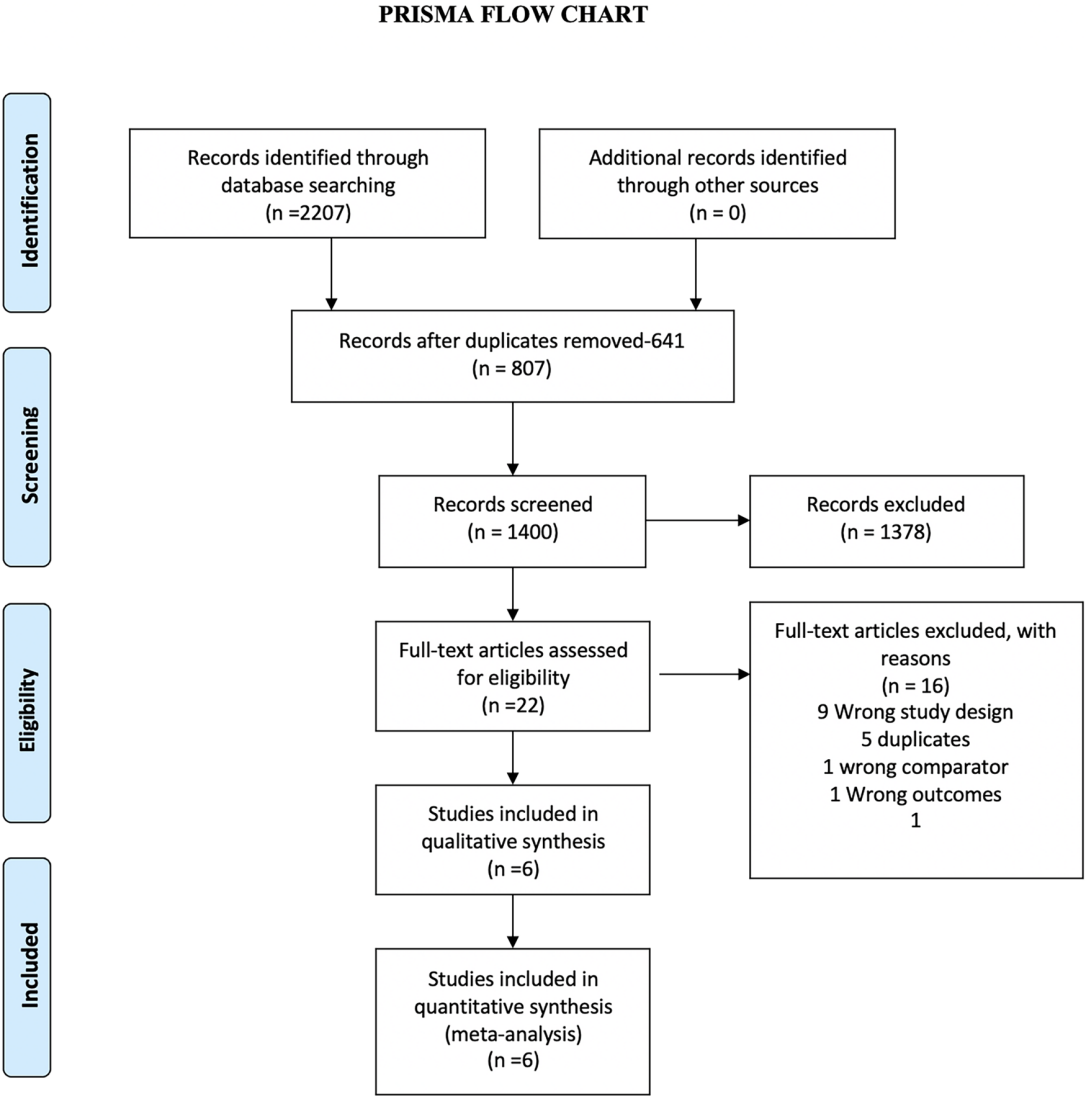
The preferred reporting items for systematic reviews and meta-analyses (PRISMA) Chart. Electronic search from databases and study selection.

We identified 2,207 studies through our databases search (Figure 1 and Supplementary Table S1). After removing 807 duplicate results, we selected 1,400 articles for title and abstract screening. We excluded 1,378 publications which were irrelevant. Twenty-two articles were studied for eligibility. Out of 22 studies, 15 were excluded and 6 studies were included for the final analysis. The reasons for exclusion of 16 studies are illustrated in Figure 1.

### 3.1. Characteristics of included studies

**Figure 2:**
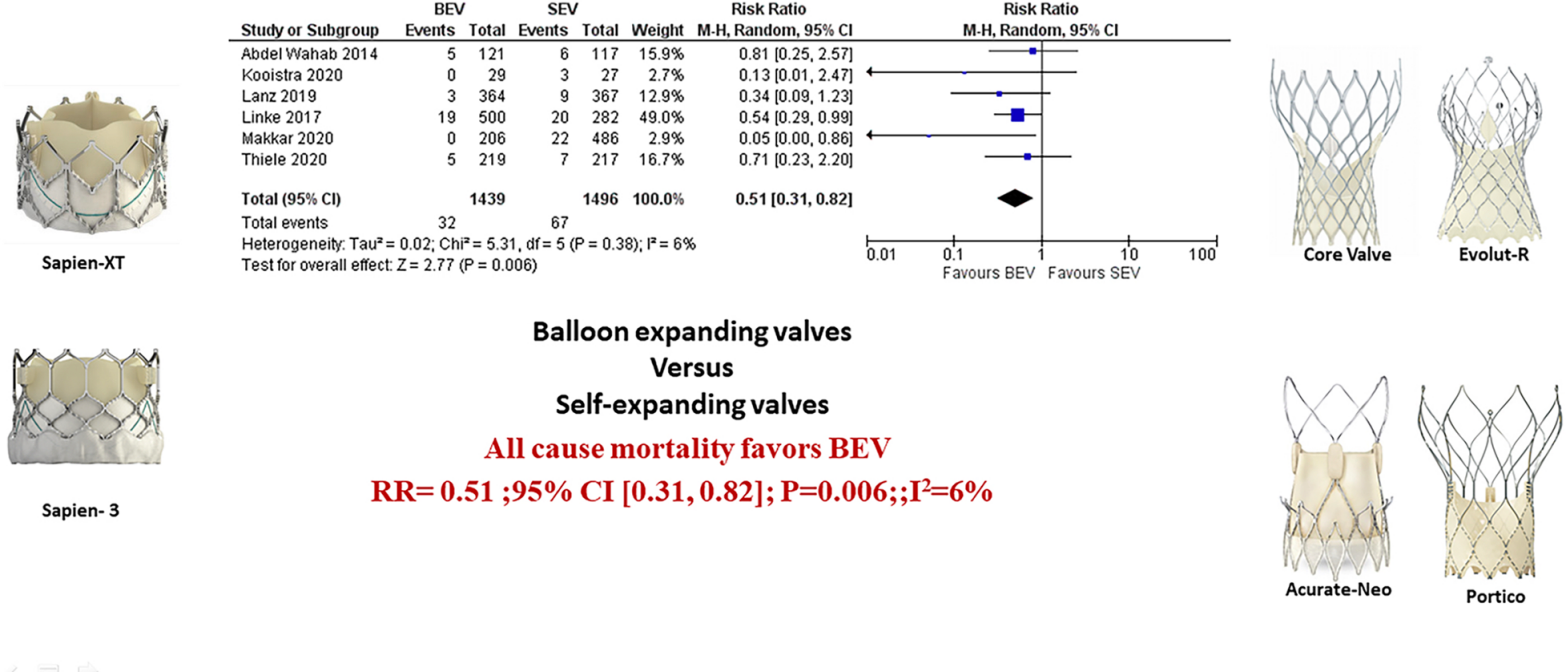
Comparison of balloon expandable platform vs. self-expanding platforms in high risk patients undergoing TAVR, BEV is associated with reduced risk of all-cause mortality at 30 days compared with SEV (**A**). The sub-group difference was not significant when the studies were stratified for the type of study (**B**). BEV, balloon expandable valve; SEV, self-expanding valve; TAVR, transcatheter aortic valve replacement; M-H, Mantel-Haenszel; CI, confidence interval.

Six studies were included for final qualitative synthesis, of which four were RCTs (10–13), two were *post hoc* analysis of RCTs (14, 15) and one was a pre-specified device or valve type analysis of an RCT investigating the different peri-procedural antithrombotic strategies in patients undergoing TAVR (15) (Table 1). The *post-hoc* study belonged to the Portico-IDE (investigational device exemption) randomized trial, presented at international conferences (14, 15) but was not yet published in a peer-reviewed at the time of the databases search. Risk of-bias as assessed by RoB-2, showed low risk for 4 studies, some concern for 1 study and high risk for one study (Supplementary Table S2). BEV (Old generation-Sapien-XT and new generation- Sapien-3) was studied against four different types of SEVs (old generation SEV CoreValve and new generation SEVs that included Evolut R, Evolut PRO, Acurate neo, and Portico) and included a total of 3,141 patients (Figure 2). Out of 2,935 patients, 1,439 patients (49.0%) received BEV and 1,496 patients (51%) received SEV. The mean age of the patient population was 81.9 years and 53.3% were female. Baseline characteristics of included studies are summarized in the Supplementary Table S3.

**Table 1:** Characteristics of trials included in the metanalysis^a^.

Characteristics	Study 1	Study 2	Study 3	Study 4	Study 5	Study 6
Study (year)	Abdel-Wahab 2014	Kooistra 2020	Lanz 2019	Linke 2017	Makkar 2020	Thiele 2020
Study design	RCT parallel group	RCT parallel group	RCT parallel group	RCT with prespecified analysis	RCT with *post-hoc* analysis	RCT parallel group
Study period	March 2012 and December 2013	January 2014 and May 2016	February 8, 2017, and February 2, 2019,	October 2012 and May 2015	May 2014 to October 2017	April 2016 to April 2018
*N*	241	56	731	782	692	438
Clinical risk	High risk	High or inoperable	Increased or inoperable	High	High or extreme	High
TAVR valve type	BEV (Sapien XT) vs. SEV (Corevalve)	BEV (Sapien 3) vs. SEV (Corevalve)	BEV (Sapien 3) vs. Acurate neo (SEV)	BEV (Sapien XT, Sapien 3) vs. SEV (Corevalve (83%), Evolut R (17%), Other non BEV was used in 5 patients)	BEV (Sapien 3) vs. SEV (Evolut R/Pro/Portico)	BEV (Sapien 3) vs. SEV (Evolut R)
Primary endpoint	Device success	Severity of post-procedural AR, quantitatively assessed by MRI	The primary composite safety and efficacy endpoint the procedure^b^	Co-primary outcome was 30-day net adverse cardiac events, NACE, composite of major adverse cardiovascular events, MACE [all-cause mortality, myocardial infarction (MI), or stroke] or major bleeding.	Hemodynamics, paravalvular aortic regurgitation, patient prosthesis mismatch (PPM) and clinical outcomes	The primary efficacy composite endpoint^c^

^a^ All included studies have reported all-cause mortality at 30 days as per our inclusion criteria.

^b^ Comprised all-cause death, any stroke, life-threatening or disabling bleeding, major vascular complications, coronary artery obstruction requiring intervention, acute kidney injury (stage 2 or 3), rehospitalisation for valve-related symptoms or congestive heart failure, valve-related dysfunction requiring repeat procedure, moderate or severe prosthetic valve regurgitation, or prosthetic valve stenosis within 30 days.

^c^ Composite endpoint of all-cause mortality, stroke, moderate/severe prosthetic valve regurgitation, and permanent pacemaker implantation at 30 days was powered for equivalence.

### 3.2. Clinical outcomes in the patient population

**Figure 3:**
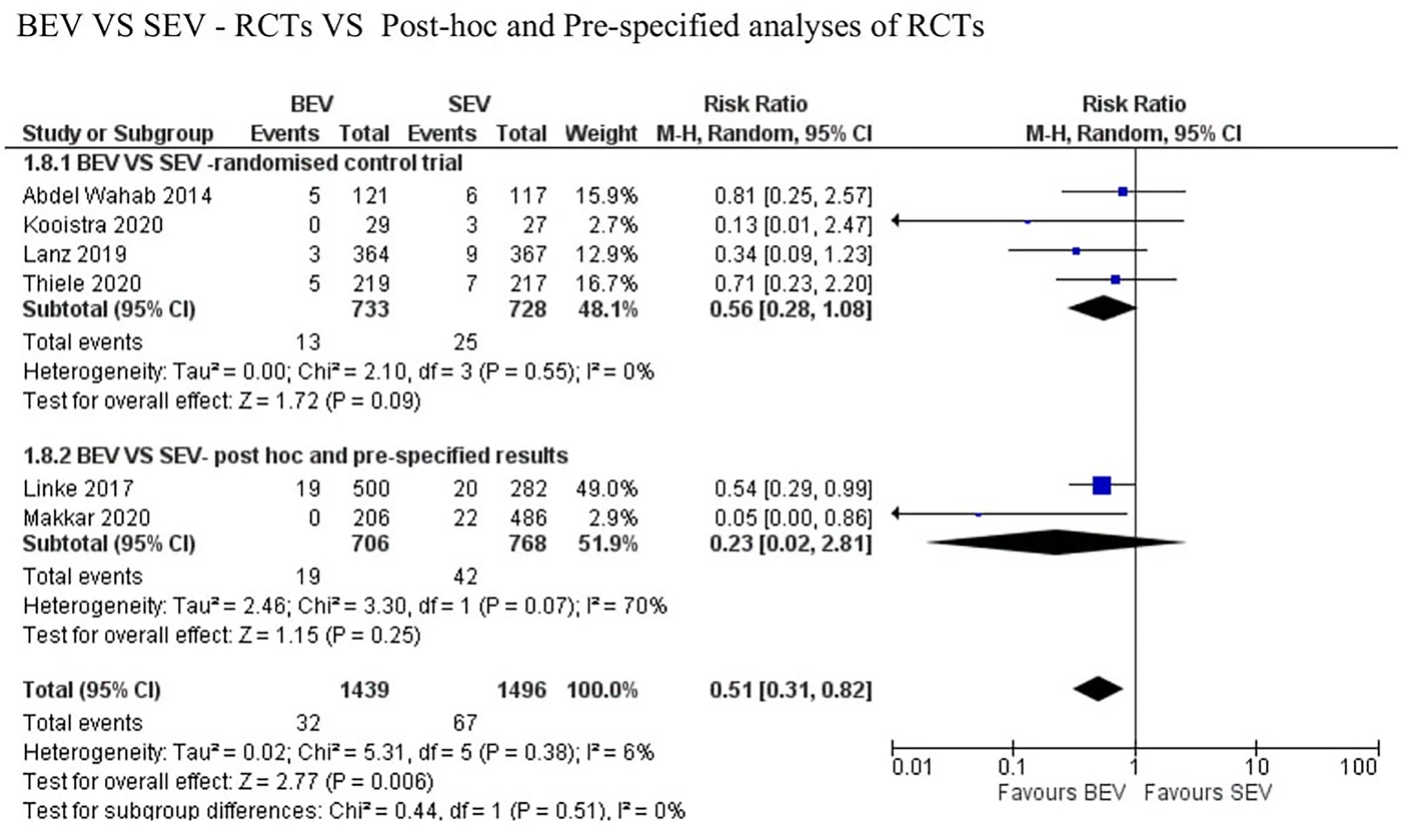
Comparison of balloon expandable platform with old generation self-expanding platforms for to assess the role of study type (RCTs comparing BEV vs. SEV head to head with *post-hoc* and pre-specified analyses of RCTs) showing no between-the-group difference on the result. BEV, balloon expandable valve; SEV, self-expanding valve; TAVR, trans-catheter aortic valve replacement; M-H, Mantel-Haenszel; CI, confidence interval.

Data regarding all-cause mortality was available from all six studies. Out of 1,439 patients who received BEV, 32 patients (2.2%) died at 30-day follow-up. In the SEV group, 67 patients (4.5%) died out of 1,496 patients. BEV was associated with significantly lower risk of death at 30 days compared with SEV [(2.2% vs. 4.5%; RR: 0.51; 95% CI: 0.31–0.82; *p* < 0.006, *I*^2^ = 6%); High level of GRADE Evidence); (Central Illustration, Figure 2)]. Sub-group analysis was performed showed no effect of new generation SEV (Evolut-R, Acurate-Neo and Portico) vs. old generation SEV (CoreValve) on the result (Supplementary Figure S1). A sensitivity analysis using Mantel-Haenszel methods using a fixed effect model showed similar result at 30 days (*p* < 0.0001) (Supplementary Figure S2). Further sensitivity analyses were performed by leave-one out statistical analyses showed similar results (Supplementary Figure S3). Additional analyses based on the role of study type (RCTs comparing BEV vs. SEV head to head with *post-hoc* and pre-specified analyses of RCTs), and the role of recapturable valves revealed no significant subgroup effects (Figure 3 and Supplementary Figure S4, respectively). There was no publication bias as assessed by funnel plot (Supplementary Figure S5).

**Figure 4:**
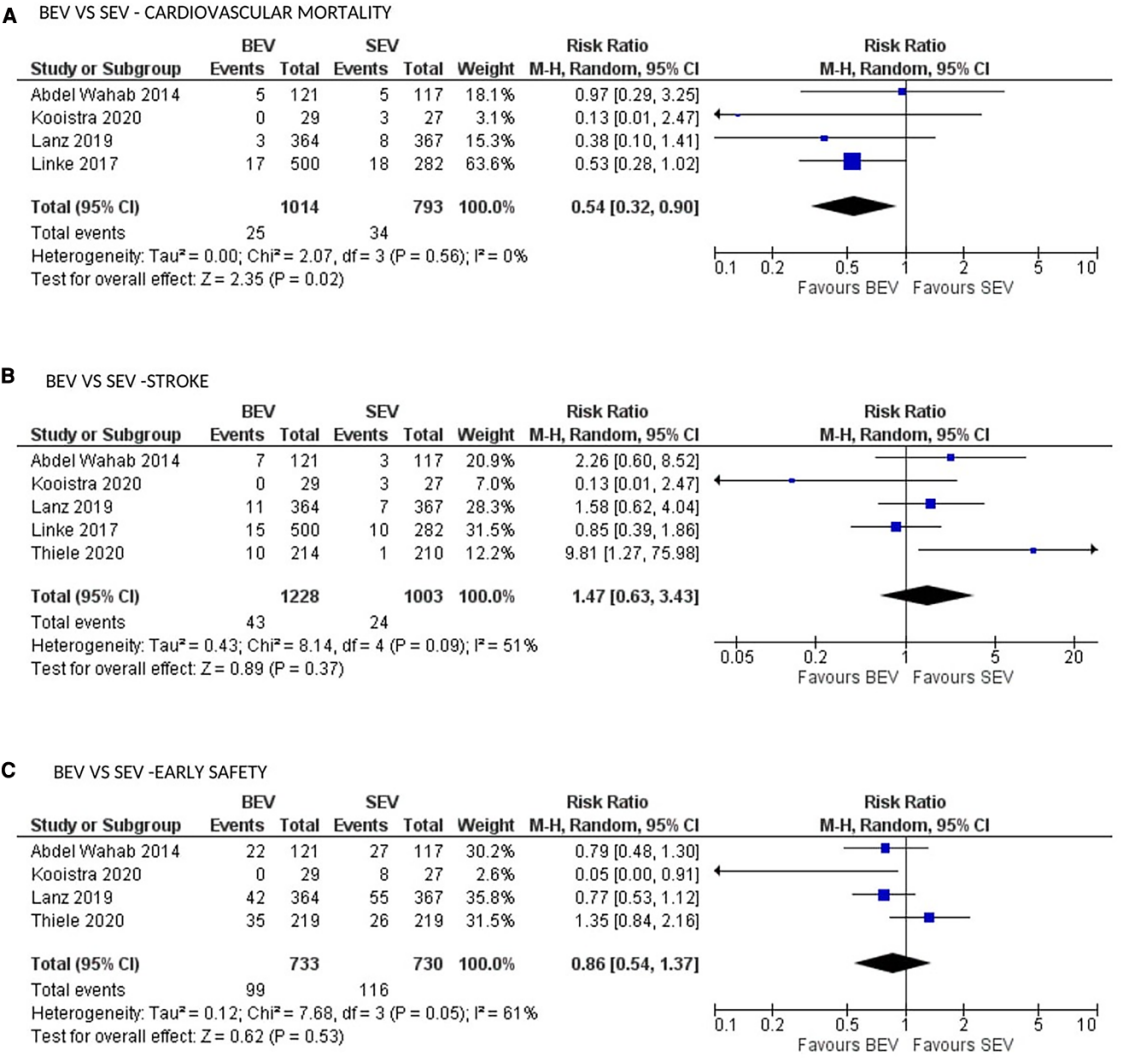
Comparison of balloon expandable platform vs. self-expanding platforms for cardiovascular mortality (**A**), stroke (**B**) and early safety (**C**) at 30 days in high risk patients undergoing TAVR. BEV, balloon expandable valve; SEV, self-expanding valve; TAVR, transcatheter aortic valve replacement; M-H, Mantel-Haenszel; CI, confidence interval.

**Figure 5:**
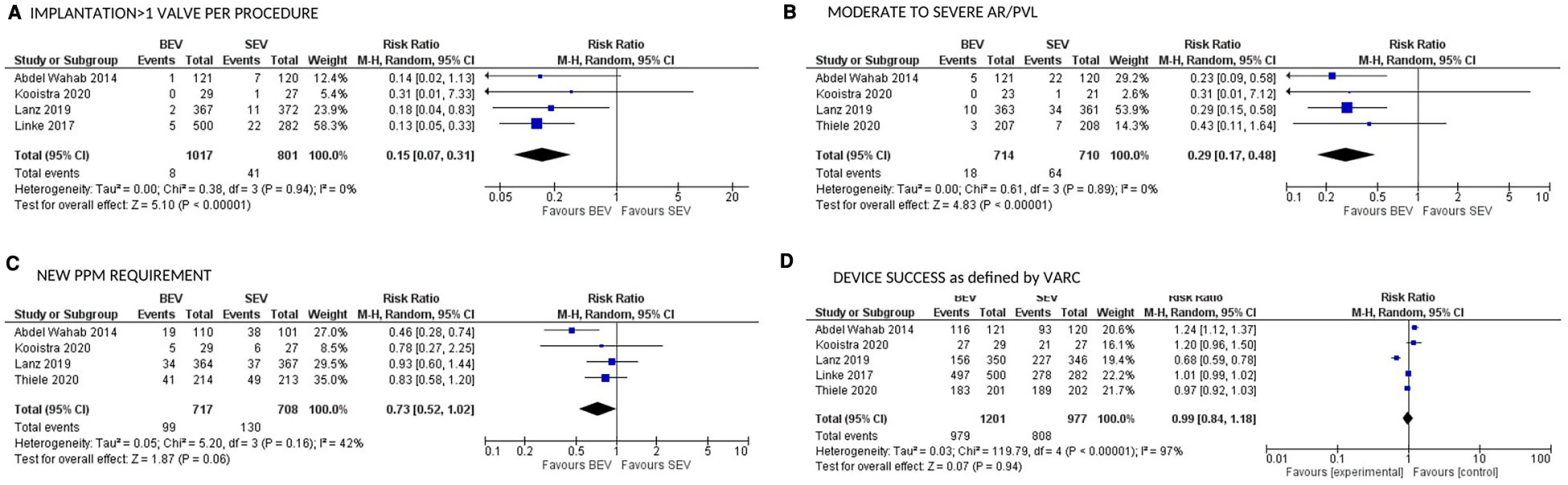
Comparison of balloon expandable platform vs. self-expanding platforms for implantation of >1 valve per procedure (**A**), moderate to severe AR/PVL (**B**), new PPM requirement (**C**), and device success as defined by VARC (**D**) at 30 days in high risk patients undergoing TAVR. BEV, balloon expandable valve; SEV, self-expanding valve; AR, aortic regurgitation; PVL, para valvular leak; PPM, permanent pacemaker; TAVR, transcatheter aortic valve replacement; VARC, valve academic research consortium; M-H, Mantel-Haenszel; CI, confidence interval.

CV mortality at 30 days was available in 5 studies. BEV was associated with lower 30-day CV mortality compared with SEV [(2.5% vs. 4.3%; RR: 0.54; 95% CI: 0.32–0.90; *p* = 0.02; *I*^2^ = 0%; Very low level of GRADE Evidence) (Figure 4A)]. Implantation of more than one valve per procedure (0.8% vs. 5.1%; RR: 0.15; 95% CI: 0.07–0.31); *p* < 0.00001; *I*^2^ = 0%; moderate level of GRADE Evidence), and moderate/severe AR/PVL (2.5% vs. 9.0%; RR: 0.29; 95% CI: 0.17–0.48); *p* < 0.00001; *I*^2^ = 0%; high level of GRADE Evidence) were also lower in the BEV arm (Figures 5A,B). A trend of reduced usage of pacemaker was observed with BEV as compared with SEV [(13.8% vs. 18.3%; RR: 0.73; 95% CI: 0.52–1.02; *p* = 0.06; *I*^2^ = 42%; very low level of GRADE Evidence) Figure 5C].

**Figure 6:**
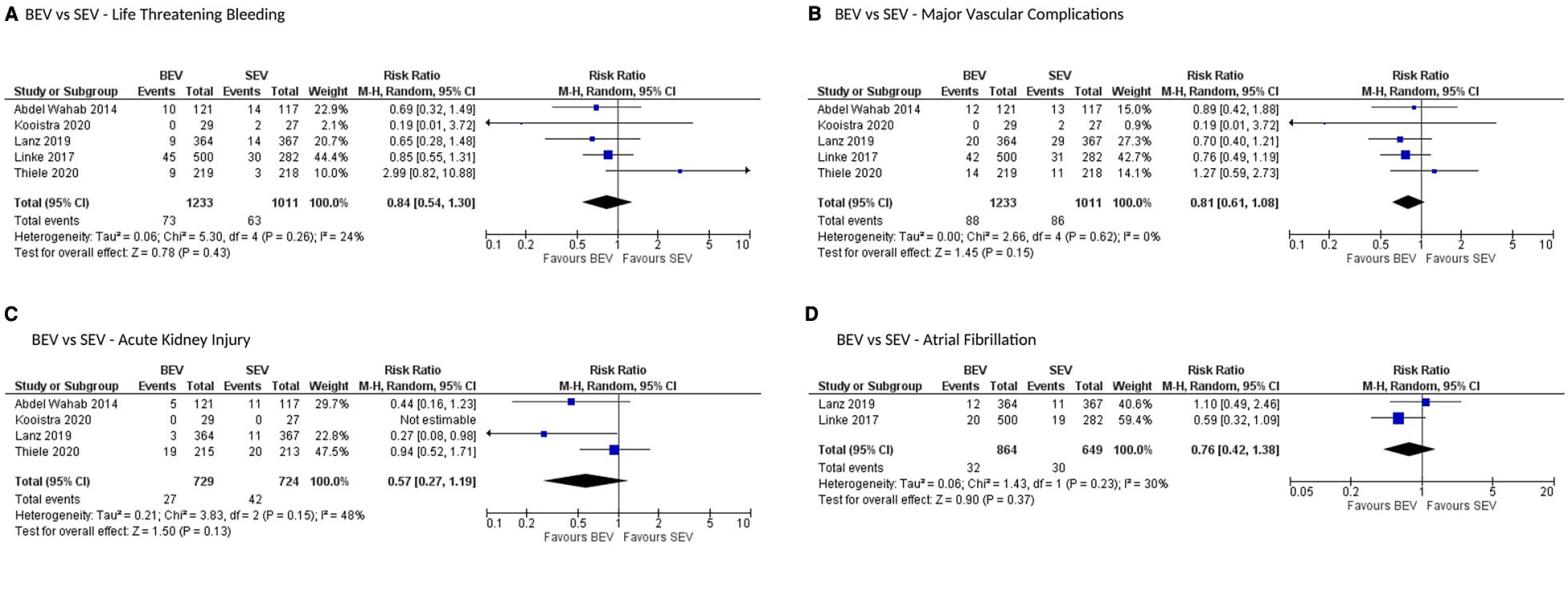
Comparison of balloon expandable platform vs. self-expanding platforms for life-threatening bleeding (**A**), major vascular complications (**B**), acute kidney injury (**C**), and atrial fibrillation (**D**) at 30 days in high risk patients undergoing TAVR. BEV, balloon expandable valve; SEV, self-expanding valve; TAVR, transcatheter aortic valve replacement; M-H, Mantel-Haenszel; CI, confidence interval.

All stroke (disabling and non-disabling) data were available in 6 studies. No significant difference in all stroke at 30 days was observed (Figure 4B). Similarly, no significant difference was noted between the 2 groups for early safety outcome at 30 days (Figure 4C), device success (Figure 5D), life-threatening bleeding, major vascular complications, AKI and atrial fibrillation (Figures 6A–D). In addition, no significant difference was found between the study arms for MI, major bleeding, rehospitalizations for valve-related symptoms or worsening congestive heart failure, valve related dysfunction requiring repeat procedure, valve malposition, and clinical efficacy (Supplementary Figures S6–S11). Sensitivity analysis using fixed effect model showed no difference in all the above parameters except AKI which was significantly lower in patients receiving BEV, with moderate heterogeneity (RR: 0.64; 95% CI: 0.40–1.01); *p* = 0.06; *I*^2^ = 48%) (Supplementary Table S4). Outcomes on cardiac tamponade, annular rupture, NYHA class improvement, NYHA status ≥class 3, and conversion to open heart surgery and valve related dysfunction were not analyzed as their reported numbers were very low.

### 3.3. Risk of bias assessment and quality of evidence

Four out of six studies had only low risk of bias assessment as assessed by Cochrane risk-of-bias tool for randomized trials version 2 (RoB 2). Two studies had some concern and one study was deemed to have risk of bias. GRADE system-based quality assessment was done for individual outcomes and a “Summary of findings” and GRADE “Evidence profile” are discussed in the Supplementary Tables S5, S6 respectively.

## 4. Discussion

In this meta-analysis of RCTs evaluating different THV platforms in high risk TAVR patients, BEV was associated with a lower risk of all-cause mortality at 30 days. In addition, BEV was found to be associated with lower CV mortality, reduced need for implantation of more than one valve per procedure, trend of decreased need for permanent pacemaker, and a lower incidence of moderate- severe AR/PVL at 30 days. There was no difference between BEV and SEV in terms of all stroke, MI, life-threatening bleeding, major vascular complications, rehospitalizations for valve-related symptoms or worsening congestive heart failure, valve related dysfunction requiring repeat procedure, valve malposition, AKI, atrial fibrillation, device success, early safety and clinical efficacy at 30 days. To our knowledge, this is the first systematic review and meta-analysis comparing BEV vs. all SEV platforms. The strengths of our systematic review include inclusion of RCTs only (including *post hoc* analysis or pre-specified analysis), detailed assessment of risk of-bias and rating the certainty of evidence utilizing the GRADE approach for all outcomes.

Only few studies have compared the safety and efficacy of BEV vs. SEV. The CENTER collaboration investigators studied 4,096 pairs of patients using propensity score matching from a pool of 12,381 TAVR patients (17). They observed lower in-hospital mortality in the BEV arm compared with the SEV arm (RR = 0.8; 95% CI: 0.6–0.9; *p* = 0.009); however, no difference was seen in 30 day-mortality [5.3% vs. 6.2%; relative risk, 0.9 (95% CI: 0.7–1.0); *p *= 0.10]. Similarly, reduced incidence of stroke (*p *= 0.03) and pacemaker requirement (*p* < 0.001) was noted in BEV. In contrast to our meta-analysis which included only RCTs, CENTER collaboration aggregated data from 9 registries and only one RCT. A recently published Bayesian meta-analysis comparing BEV vs. SEV found no difference in all-cause mortality, CV mortality, stroke, PVL, vascular complications but showed less pacemaker implantation with BEV (18). In contrast to our study, Osman et al. included 8 RCTs of all risk categories, out of which only one was a RCT comparing BEV with SEV in a head-to-head fashion. Four more studies have been published or presented comparing BEV with SEV using three different platforms after the above meta-analysis. Hence, an appropriately conducted pair-wise meta-analysis is warranted to compare the safety and efficacy of BEV vs. SEV.

Two RCTs comparing BEV vs. SEV were recently published. In SCOPE-1, 739 high risk patients were randomized to receive Acurate-Neo vs. Sapien 3. SEV failed to meet its non-inferiority for primary safety and clinical efficacy composite endpoint at 30 days (12). SOLVE-TAVI compared SEV (Evolut R) with BEV (Sapien-3) (13) among 447 high risk patients with aortic stenosis undergoing TF TAVR. No difference in all-cause mortality was observed at 30 days (*p* valve for equivalence <0.0001). A recently presented *post-hoc* analysis of PORTICO-IDE RCT (14, 15) comparing Portico SEV vs. commercially available Sapien-3 and Evolut R showed lower mortality in BEV at 30 days (Log-rank *p* = 0.005). The fourth RCT compared SEV with Lotus (MEV) (19), which is not within the scope of this meta-analysis.

Our study findings were also similar to those observed in two large registry analysis comparing BEV vs. SEV (20, 21). In the prospective FRANCE-TAVI registry, 3,910 matched pairs were studied from a pool of 12,141 patients. In-hospital mortality was higher in patients with SEV as compared with BEV (matched RR: 1.33; 95% CI: 1.06–1.16); *p* = 0.01). SEV was also associated with increase in moderate PVL, implantation of >1 device per procedure and permanent pacemaker implantation, although the predominant SEV used was the first-generation CoreValve. In another propensity score matched nationwide analysis from France, 10,459 patients who had received BEV (Sapien-3) or SEV (Evolut-R) were studied. Patients who had BEV had lower 1 year all-cause mortality (RR: 0.88; 95% CI: 0.82–0.95; *p* = 0.001), CV death (RR: 0.82; 95% CI: 0.73–0.92; *p* 0.0004), and rehospitalization for heart failure (RR: 0.84; 95% CI: 0.78–0.90; *p* < 0.0001) compared with SEV.

In our meta-analysis, TF-TAVR using BEV is associated with a 51% relative risk reduction in all-cause mortality and a 42% relative risk reduction in CV mortality compared with SEV arm. Test for sub-group difference was not significant based on the role of recapturable SEV or generation of SEV. The observed survival benefit with BEV could be due to their mode of deployment which is quicker, the utility of a flex-catheter in the BEV, decreased occurrence of moderate to severe AR/PVL, and reduced need for implantation of more than one valve per procedure. The use of flexible FlexNAV catheter in the parallel cohort of Portico-IDE showed similar results as compared with the BEV (15). Moderate or severe AR/PVL has been consistently shown to be associated with higher short-term and long-term mortality (22, 23). In our study, BEV was associated with a 29% relative risk reduction in moderate to severe AR/PVL. This could explain the lower 30-day mortality observed with BEV in our study. Use of a second valve was more common in SEV. However, its association with mortality could not be assessed due to non-availability of patient-level data. Increased need for permanent pacemaker observed in our study with SEV is consistent with current literature (24). The long-term implication of increased pacemaker requirement is yet to be studied and has to be considered as a significant factor, given expanded use of TAVR in low risk patients.

### 4.1. Limitations

Our limitations include being a study-level meta-analysis and not a patient-level meta-analysis. Comparison of all forms of SEV together might be a limitation in our study given different design characteristics, but all the studied SEV are made of nitinol. Two of them were recapturable (Portico and Evolut R). Sensitivity analyses investigating the impact of recapturable SEVs showed no significant effect on outcomes (Supplementary Figure S5) (25). Inclusion of *post-hoc* analyses and RCTs with pre-specified endpoints are possible limitations but sensitivity analysis showed no effect on our primary outcome. We limited our analysis to 30-day outcomes due to absence of data across trials. As the outcomes of intention to treat analyses were not available for the *post hoc* studies, we collected data from the utilized modified-as-treated analyses as reported by the authors. Some studies also excluded patients with heavy calcification in the aortic annulus, left ventricular outflow tract (LVOT) or sinotubular junction, limiting the interpretation of these findings to those subgroups. Being a study-level metanalysis, we could not calculate the outcomes based on recently published VARC-3 crtieria (26).

### 4.2. Conclusion

Balloon-expandable TAVR is associated with reduced all-cause mortality (High level of GRADE evidence), CV mortality (Very low level of GRADE evidence) at 30 days compared with self- expanding TAVR in high risk patients undergoing TF-TAVR. Longer-term data are necessary to determine if the difference in outcomes persist and if the same effects are seen in lower risk patients.

## Data Availability

The original contributions presented in the study are included in the article/**Supplementary Material**, further inquiries can be directed to the corresponding author.
