# Corrigendum: The safety and efficacy of balloon-expandable versus self-expanding trans-catheter aortic valve replacement in high-risk patients

**DOI:** 10.3389/fcvm.2023.1282812

**Published:** 2023-10-13

**Authors:** Nagendra Boopathy Senguttuvan, Hemal Bhatt, Vinod Kumar Balakrishnan, Parasuram Krishnamoorthy, Sunny Goel, Pothireddy M. K. Reddy, Vinodhini Subramanian, Bimmer E. Claessen, Ashish Kumar, Monil Majmundar, Richard Ro, Stamatios Lerakis, Ramamoorthi Jayaraj, Ankur Kalra, Marcus Flather, George Dangas

**Affiliations:** ^1^ Department of Cardiology, Sri Ramachandra Institute of Higher Education and Research, Chennai, India; ^2^ Department of Cardiology, The Zena and Michael A. Wiener Cardiovascular Institute, Icahn School of Medicine at Mount Sinai, New York, NY, United States; ^3^ Department of Cardiology, Hackensack Meridian Health, New Jersey, NJ, United States; ^4^ Department of Cardiology, Amsterdam University Medical Centres, Amsterdam, the Netherlands; ^5^ Department of Internal Medicine, Cleveland Clinic Akron General, Akron, OH, United States; ^6^ Department of Internal Medicine, New York Medical College, Metropolitan Hospital, New York, NY, United States; ^7^ Jindal Institute of Behavioral Sciences (JIBS), Jindal Global Institution of Eminence Deemed to Be University, Sonipat, India; ^8^ Department of Cardiovascular Medicine, Franciscan Health, Indiana, IN, USA; Co-CEO, Kalra Hospitals, New Delhi, India; ^9^ Professor of Cardiology, Norwich Medical School, University of East Anglia, Norwich, United Kingdom

**Keywords:** aortic stenosis, balloon expandable, self-expanding, trans catheter aortic valve replacement, valve

A Corrigendum on The safety and efficacy of balloon-expandable versus self-expanding trans-catheter aortic valve replacement in high-risk patients with severe symptomatic aortic stenosis By Senguttuvan NB, Bhatt H, Balakrishnan VK, et al. (2023) Front. Cardiovasc. Med. 10:1130354. doi: 10.3389/fcvm.2023.1130354

**Error in Author List**

In the published article, there was an error in the author list. The previously-named author Gilbert H. L. Tang needs to be removed. The corrected author list appears below.

Nagendra Boopathy Senguttuvan^1,2^, Hemal Bhatt^2,3^, Vinod Kumar Balakrishnan^1^, Parasuram Krishnamoorthy^3^, Sunny Goel^3^, Pothireddy M K Reddy^1^, Vinodhini Subramanian^1^, Bimmer E Claessen^2,4^, Ashish Kumar^5^, Monil Majmundar^6^, Richard Ro^2^, Stamatios Lerakis^2^, Ramamoorthi Jayaraj^7^, Ankur Kalra^5,8^, Marcus Flather^9^, George Dangas^2^

The authors apologize for this error and state that this does not change the scientific conclusions of the article in any way. The original article has been updated.

**Text Correction**

In the published article, there was an error. The Author Contribution section stated that Gilbert H. L. Tang's (GT) made a contribution to the article.

A correction has been made to the Author contribution section. This section previously stated:

“NS: planning, execution, data extraction, statistics, reporting, writing the first manuscript, review and editing. HB: planning, execution, data extraction, statistics. VB, PK, SG, RR, SL, AKu, MM, RJ, Aka, MF, GD, and VS: review and editing. PR: data extraction, review and editing. BC: planning, execution, review and editing. GT: senior author, guarantor.”

The corrected section appears below.

“NS: planning, execution, data extraction, statistics, reporting, writing the first manuscript, review and editing. HB: planning, execution, data extraction, statistics. VB, PK, SG, RR, SL, AKu, MM, RJ, Aka, MF, GD, and VS: review and editing. PR: data extraction, review and editing. BC: planning, execution, review and editing.”

The authors apologize for this error and state that this does not change the scientific conclusions of the article in any way. The original article has been updated.

