# Supplementary material for: The safety and efficacy of balloon-expandable versus self-expanding trans-catheter aortic valve replacement in high-risk patients with severe symptomatic aortic stenosis
Source: Front Cardiovasc Med. 2023 May 25;10:1130354. doi: 10.3389/fcvm.2023.1130354 (PMC10283153; doi:10.3389/fcvm.2023.1130354)
Supplement: Supplementary file 1 [file Datasheet1.docx]

***Supplementary Material***

**Article Title:** The Safety and Efficacy of Balloon-Expandable Versus Self- Expanding Trans-catheter Aortic Valve Replacement in High- Risk Patients

1. **Supplementary Data**

**Key words used for search strategy:**

(aortic stenos* or Aortic Valve Stenos*) and (Transcatheter Aortic Valve replacement or Transcatheter Aortic Valve Implantation or TAVR or TAVI or percutaneous aortic valve replacement or transfemoral aortic valve replacement) and (self expanding or balloon expanding or SEV or BEV or Core Valve or CoreValve or Evolut R or Evolut PRO or Edwards sapien or sapien XT or Sapien 3 or Sapien 3 Ultra or Acurate or AcurateNeo or Jena valve or centera or Venus A-valve) and (Randomized)

**Databases searched:**

**Pubmed, Embase, Scopus, Clinicaltrials.gov, and Web of Sciences.­**

**EMBASE search:**

(aortic stenos* or Aortic Valve Stenos*).mp. and (Transcatheter Aortic Valve replacement or Transcatheter Aortic Valve Implantation or TAVR or TAVI or percutaneous aortic valve replacement or transfemoral aortic valve replacement).mp. and (self expanding or balloon expanding or SEV or BEV or Core Valve or CoreValve or Evolut R or Evolut PRO or Edwards sapien or sapien XT or Sapien 3 or Sapien 3 Ultra or Acurate or AcurateNeo or Jena valve or centera or Venus A-valve).mp. and (Randomized).mp.

**PubMed search:**

("Aortic Valve Stenosis"[Mesh] OR aortic stenos* OR Aortic Valve Stenos*) AND ("Transcatheter Aortic Valve Replacement"[Mesh] OR Transcatheter Aortic Valve replacement OR Transcatheter Aortic Valve Implantation OR TAVR OR TAVI OR percutaneous aortic valve replacement OR transfemoral aortic valve replacement) AND (self expanding OR balloon expanding OR SEV OR BEV OR Core Valve OR CoreValve OR Evolut R OR Evolut PRO OR Edwards sapien OR sapien XT OR Sapien 3 OR Sapien 3 Ultra OR Acurate OR AcurateNeo OR Jena valve OR centera or Venus A-valve) AND (Randomized)

**Scopus search (401 results on April 16, 2020):**

TITLE-ABS-KEY(((aortic stenos*) OR (Aortic Valve Stenos*))) **AND** TITLE-ABS-KEY(((Transcatheter Aortic Valve replacement) OR (Transcatheter Aortic Valve Implantation) OR TAVR OR TAVI OR (percutaneous aortic valve replacement) OR (transfemoral aortic valve replacement))) **AND** TITLE-ABS-KEY(((self expanding) OR (balloon expanding) OR SEV OR BEV OR (Core Valve) OR CoreValve OR (Evolut R) OR (Evolut PRO) OR (Edwards sapien) OR (sapien XT) OR (Sapien 3) OR Acurate OR AcurateNeo OR (Jena valve) OR centera OR (Venus A-valve))) **AND** TITLE-ABS-KEY((Randomized))

**Web of Science search (177 results on April 16, 2020):**

TS=((aortic stenos*) OR (Aortic Valve Stenos*))

TS=((Transcatheter Aortic Valve replacement) OR (Transcatheter Aortic Valve Implantation) OR TAVR OR TAVI OR (percutaneous aortic valve replacement) OR (transfemoral aortic valve replacement))

TS=((self expanding) OR (balloon expanding) OR SEV OR BEV OR (Core Valve) OR CoreValve OR (Evolut R) OR (Evolut PRO) OR (Edwards sapien) OR (sapien XT) OR (Sapien 3) OR Acurate OR AcurateNeo OR (Jena valve) OR centera OR (Venus A-valve))

TS=(Randomized)

**ClinicalTrials.gov (319 results on April 16, 2020)**

Condition or disease: aortic stenosis

Other terms: transcatheter

1. **Supplementary Figures and Tables**
   1. **Supplementary tables**

**Table S1.** PRISMA Checklist

| **Section/topic** | **#** | **Checklist item** | **Reported on page #** |
| --- | --- | --- | --- |
| **TITLE** | | |  |
| Title | 1 | Identify the report as a systematic review, meta-analysis, or both. | 1 |
| **ABSTRACT** | | |  |
| Structured summary | 2 | Provide a structured summary including, as applicable: background; objectives; data sources; study eligibility criteria, participants, and interventions; study appraisal and synthesis methods; results; limitations; conclusions and implications of key findings; systematic review registration number. | 2,3 |
| **INTRODUCTION** | | |  |
| Rationale | 3 | Describe the rationale for the review in the context of what is already known. | 5 |
| Objectives | 4 | Provide an explicit statement of questions being addressed with reference to participants, interventions, comparisons, outcomes, and study design (PICOS). | 5 |
| **METHODS** | | |  |
| Protocol and registration | 5 | Indicate if a review protocol exists, if and where it can be accessed (e.g., Web address), and, if available, provide registration information including registration number. | CRD42020181190 |
| Eligibility criteria | 6 | Specify study characteristics (e.g., PICOS, length of follow-up) and report characteristics (e.g., years considered, language, publication status) used as criteria for eligibility, giving rationale. | 6 |
| Information sources | 7 | Describe all information sources (e.g., databases with dates of coverage, contact with study authors to identify additional studies) in the search and date last searched. | 6 & Supplementary document 1 |
| Search | 8 | Present full electronic search strategy for at least one database, including any limits used, such that it could be repeated. | Supplementary document 1 |
| Study selection | 9 | State the process for selecting studies (i.e., screening, eligibility, included in systematic review, and, if applicable, included in the meta-analysis). | 6 |
| Data collection process | 10 | Describe method of data extraction from reports (e.g., piloted forms, independently, in duplicate) and any processes for obtaining and confirming data from investigators. | 7 |
| Data items | 11 | List and define all variables for which data were sought (e.g., PICOS, funding sources) and any assumptions and simplifications made. | 7-8 |
| Risk of bias in individual studies | 12 | Describe methods used for assessing risk of bias of individual studies (including specification of whether this was done at the study or outcome level), and how this information is to be used in any data synthesis. | 11 & Supplementary document 9-14 |
| Summary measures | 13 | State the principal summary measures (e.g., risk ratio, difference in means). | 7-8 |
| Synthesis of results | 14 | Describe the methods of handling data and combining results of studies, if done, including measures of consistency (e.g., I^2^) for each meta-analysis. | 8 |

| **Section/topic** | **#** | **Checklist item** | **Reported on page #** |
| --- | --- | --- | --- |
| Risk of bias across studies | 15 | Specify any assessment of risk of bias that may affect the cumulative evidence (e.g., publication bias, selective reporting within studies). | 11 & Supplementary document 9-14 |
| Additional analyses | 16 | Describe methods of additional analyses (e.g., sensitivity or subgroup analyses, meta-regression), if done, indicating which were pre-specified. | Supplementary Pages 18-36 |
| **RESULTS** | | |  |
| Study selection | 17 | Give numbers of studies screened, assessed for eligibility, and included in the review, with reasons for exclusions at each stage, ideally with a flow diagram. | Figure 1 |
| Study characteristics | 18 | For each study, present characteristics for whi­­ch data were extracted (e.g., study size, PICOS, follow-up period) and provide the citations. | 25 ;Table 1; Supplementary Pages 15-17 |
| Risk of bias within studies | 19 | Present data on risk of bias of each study and, if available, any outcome level assessment (see item 12). | Supplementary document 9-14 |
| Results of individual studies | 20 | For all outcomes considered (benefits or harms), present, for each study: (a) simple summary data for each intervention group (b) effect estimates and confidence intervals, ideally with a forest plot. | Figure 2-5  And Supplementary document Figure S1-S11(18-36) |
| Synthesis of results | 21 | Present results of each meta-analysis done, including confidence intervals and measures of consistency. | 8-11  Supplementary document Figure S1-S11(18-36) |
| Risk of bias across studies | 22 | Present results of any assessment of risk of bias across studies (see Item 15). | 11 & Supplementary document 9-14 |
| Additional analysis | 23 | Give results of additional analyses, if done (e.g., sensitivity or subgroup analyses, meta-regression [see Item 16]). | Supplementary document Figure S1-S11(18-36) and Table S4 |
| **DISCUSSION** | | |  |
| Summary of evidence | 24 | Summarize the main findings including the strength of evidence for each main outcome; consider their relevance to key groups (e.g., healthcare providers, users, and policy makers). | 11-14 |
| Limitations | 25 | Discuss limitations at study and outcome level (e.g., risk of bias), and at review-level (e.g., incomplete retrieval of identified research, reporting bias). | 14-15 |
| Conclusions | 26 | Provide a general interpretation of the results in the context of other evidence, and implications for future research. | 15 |
| **FUNDING** | | |  |
| Funding | 27 | Describe sources of funding for the systematic review and other support (e.g., supply of data); role of funders for the systematic review. | 11-17 |

PRISMA = Preferred Reporting Items for Systematic Reviews and Meta-Analyses.

**Table S2.** Risk of Bias Assessment

| **Study details**   \| ***Reference*** \| 1. Abdel-Wahab M, Mehilli J, Frerker C, Neumann F-J, Kurz T, Tölg R, et al. Comparison of balloon-expandable vs self-expandable valves in patients undergoing transcatheter aortic valve replacement: the CHOICE randomized clinical trial. JAMA. 2014 Apr 16;311(15):1503–14 2. Kooistra NHM, Abawi M, Voskuil M, Urgel K, Samim M, Nijhoff F, et al. Randomised comparison of a balloon-expandable and self-expandable valve with quantitative assessment of aortic regurgitation using magnetic resonance imaging. Neth Heart J Mon J Neth Soc Cardiol Neth Heart Found. 2020 Apr 3 3. Lanz J, Kim W-K, Walther T, Burgdorf C, Möllmann H, Linke A, et al. Safety and efficacy of a self-expanding versus a balloon-expandable bioprosthesis for transcatheter aortic valve replacement in patients with symptomatic severe aortic stenosis: a randomised non-inferiority trial. Lancet Lond Engl. 2019 02;394(10209):1619–28. 4. Linke A, Chandrasekhar J, Sartori S, Lefevre T, van Belle E, Schaefer U, et al. Effect of valve design and anticoagulation strategy on 30-day clinical outcomes in transcatheter aortic valve replacement: Results from the BRAVO 3 randomized trial. Catheter Cardiovasc Interv Off J Soc Card Angiogr Interv. 2017 Nov 15;90(6):1016–26. 5. Maisano PF. PORTICO: A Randomized Trial of Portico vs. Commercially Available Transcatheter Aortic Valves in Patients With Severe Aortic Stenosis [Internet]. TCTMD.com. [cited 2020 Apr 29]. Available from: https://www.tctmd.com/slide/portico-randomized-trial-portico-vs-commercially-available-transcatheter-aortic-valves 6. Makkar RR, Waksman R, Groh M, Russo MJ, Hermiller J, Worthley S, et al. CRT-600.01 Comparison of Valve Performance of the Intra-Annular Self-Expanding Portico^TM^ Transcatheter Aortic Valve With Contemporary Supra-Annular Self-Expanding and Intra-Annular Balloon-Expandable Valves: Insights From the PORTICO IDE Trial. JACC Cardiovasc Interv. 2020 Feb 24;13(4 Supplement):S46. 7. Thiele H, Kurz T, Feistritzer H-J, Stachel G, Hartung P, Eitel I, et al. Comparison of newer generation self-expandable vs. balloon-expandable valves in transcatheter aortic valve implantation: the randomized SOLVE-TAVI trial. Eur Heart J. 2020 Feb 12; \| \| --- \| --- \|   **Study design**   \| ⮽ \| Individually-randomized parallel-group trial \| \| --- \| --- \| \| □ \| Cluster-randomized parallel-group trial \| \| □ \| Individually randomized cross-over (or other matched) trial \|  \| **Specify which outcome is being assessed for risk of bias** \| All-cause mortality at 30 days \| \| --- \| --- \|  \| **Specify the numerical result being assessed.** In case of multiple alternative analyses being presented, specify the numeric result (e.g. RR = 1.52 (95% CI 0.83 to 2.77) and/or a reference (e.g. to a table, figure or paragraph) that uniquely defines the result being assessed. \| RR, 0.48 ;95% CI [0.29, 0.80]  P=0.005; I^2^=12% (Central Illustration) \| \| --- \| --- \|   **Is the review team’s aim for this result…?**   \| ⮽ \| to assess the effect of *assignment to intervention* (the ‘intention-to-treat’ effect) \| \| --- \| --- \| \| □ \| to assess the effect of *adhering to intervention* (the ‘per-protocol’ effect) \|   **Which of the following sources were obtained to help inform the risk-of-bias assessment? (tick as many as apply)**  ⮽ Journal article(s) with results of the trial  ⮽ Trial protocol  ⮽ Statistical analysis plan (SAP)  ⮽ Non-commercial trial registry record (e.g. ClinicalTrials.gov record)  □ Company-owned trial registry record (e.g. GSK Clinical Study Register record)  □ “Grey literature” (e.g. unpublished thesis)  ⮽ Conference abstract(s) about the trial  □ Regulatory document (e.g. Clinical Study Report, Drug Approval Package)  □ Research ethics application  □ Grant database summary (e.g. NIH RePORTER or Research Councils UK Gateway to Research)  □ Personal communication with trialist  □ Personal communication with the sponsor |
| --- | --- | --- | --- | --- | --- | --- | --- | --- | --- | --- | --- | --- | --- | --- | --- | --- |

**Domain 1: Risk of bias arising from the randomization process**

| **Signaling questions** | Abdul Waheb et al | Kooistra et al | Lanz et al | Linke et al | Makkar et al (BEV Vs EV or Portico) | Thiele et al |
| --- | --- | --- | --- | --- | --- | --- |
| **1.1 Was the allocation sequence random?** | Y | Y | Y | N | Y | Y |
| **1.2 Was the allocation sequence concealed until participants were enrolled and assigned to interventions?** | Y | Y | Y | N | Y | Y |
| **1.3 Did baseline differences between intervention groups suggest a problem with the randomization process?** | N | N | N | **Y** | N | N |
| **Risk-of-bias judgement** | Low | Low | Low | High | Low | Low |
| Optional: What is the predicted direction of bias arising from the randomization process? | - | - | - | NA | - | - |

Domain 2: Risk of bias due to deviations from the intended interventions (*effect of assignment to intervention*)

| **Signaling questions** | Abdul Waheb et al | Kooistra et al | Lanz et al | Linke et al | Makkar et al (BEV Vs Evolut and Portico) | Thiele et al |
| --- | --- | --- | --- | --- | --- | --- |
| **2.1. Were participants aware of their assigned intervention during the trial?** | Y | Y | Y | Y | Y | Y |
| **2.2. Were carers and people delivering the interventions aware of participants' assigned intervention during the trial?** | Y | Y | Y | Y | Y | Y |
| **2.3. If Y/PY/NI to 2.1 or 2.2: Were there deviations from the intended intervention that arose because of the experimental context?** | Y | N | Y | Y | Y | Y |
| **2.4. If Y/PY to 2.3: Were these deviations from intended intervention balanced between groups?** | PN |  | PN | PN | PN | PN |
| **2.5 If N/PN/NI to 2.4: Were these deviations likely to have affected the outcome?** | PN |  | PN | PN | PN | PN |
| **2.6 Was an appropriate analysis used to estimate the effect of assignment to intervention?** | Y | Y | Y | Y | Y | Y |
| **2.7 If N/PN/NI to 2.6: Was there potential for a substantial impact (on the result) of the failure to analyze participants in the group to which they were randomized?** |  |  |  |  |  |  |
| **Risk-of-bias judgement** | Some concerns | Some concerns | Some concerns | Some concerns | Some concerns | Some concerns |
| Optional: What is the predicted direction of bias due to deviations from intended interventions? |  |  |  |  |  |  |
|  |  |  |  |  |  |  |

Domain 2: Risk of bias due to deviations from the intended interventions (*effect of adhering to intervention*)

| **Signaling questions** | Abdul Waheb et al | Kooistra et al | Lanz et al | Linke et al | Makkar et al (BEV Vs EV/Portico) | Thiele et al |
| --- | --- | --- | --- | --- | --- | --- |
| **2.1. Were participants aware of their assigned intervention during the trial?** | Y | Y | Y | Y | Y | Y |
| **2.2. Were carers and people delivering the interventions aware of participants' assigned intervention during the trial?** | Y | Y | Y | Y | Y | Y |
| **2.3. If Y/PY/NI to 2.1 or 2.2: Were important co-interventions balanced across intervention groups?** | Y | Y | Y | N | N | Y |
| **2.4. Could failures in implementing the intervention have affected the outcome?** | Y | Y | Y | Y | Y | Y |
| **2.5. Did study participants adhere to the assigned intervention regimen?** | Y | Y | Y | Y | Y | Y |
| **2.6. If N/PN/NI to 2.3 or 2.5 or Y/PY/NI to 2.4: Was an appropriate analysis used to estimate the effect of adhering to the intervention?** | Y | Y | Y | Y | Y | Y |
| **Risk-of-bias judgement** | Some concerns | Some concerns | Some concerns | Some concerns | Some concerns | Some concerns |

Domain 3: Missing outcome data

| **Signaling questions** | Abdul Waheb et al | Kooistra et al | Lanz et al | Linke et al | Makkar et al (BEV Vs EV/Portico) | Thiele et al |
| --- | --- | --- | --- | --- | --- | --- |
| **3.1 Were data for this outcome available for all, or nearly all, participants randomized?** | Y | Y | Y | Y | Y | Y |
| **3.2 If N/PN/NI to 3.1: Is there evidence that result was not biased by missing outcome data?** |  |  |  |  |  |  |
| **3.3 If N/PN to 3.2: Could missingness in the outcome depend on its true value?** |  |  |  |  |  |  |
| **3.4 If Y/PY/NI to 3.3: Do the proportions of missing outcome data differ between intervention groups?** |  |  |  |  |  |  |
| **3.5 If Y/PY/NI to 3.3: Is it likely that missingness in the outcome depended on its true value?** |  |  |  |  |  |  |
| **Risk-of-bias judgement** | Low | Low | Low | Low | Low | Low |

Domain 4: Risk of bias in measurement of the outcome

| **Signaling questions** | Abdul Waheb et al | Kooistra et al | Lanz et al | Linke et al | Makkar et al (BEV Vs EV/Portico) | Thiele et al |
| --- | --- | --- | --- | --- | --- | --- |
| **4.1 Was the method of measuring the outcome inappropriate?** | N | N | N | N | N | N |
| **4.2 Could measurement or ascertainment of the outcome have differed between intervention groups ?** | N | N | N | N | N | N |
| **4.3 If N/PN/NI to 4.1 and 4.2: Were outcome assessors aware of the intervention received by study participants ?** |  |  |  |  |  |  |
| **4.4 If Y/PY/NI to 4.3: Could assessment of the outcome have been influenced by knowledge of intervention received?** |  |  |  |  |  |  |
| **4.5 If Y/PY/NI to 4.4: Is it likely that assessment of the outcome was influenced by knowledge of intervention received?** |  |  |  |  |  |  |
| **Risk-of-bias judgement** | Low | Low | Low | Low | Low | Low |

Domain 5: Risk of bias in selection of the reported result

| Signaling questions | Abdul Waheb et al | Kooistra et al | Lanz et al | Linke et al | Makkar et al (BEV Vs EV/Portico) | Thiele et al |
| --- | --- | --- | --- | --- | --- | --- |
| **5.1 Was the trial analyzed in accordance with a pre-specified plan that was finalized before unblinded outcome data were available for analysis ?** | Y | Y | Y | Y | PN | Y |
| **Is the numerical result being assessed likely to have been selected, on the basis of the results, from...** |  |  |  |  |  |  |
| **5.2. ... multiple outcome measurements (e.g. scales, definitions, time points) within the outcome domain?** | N | N | N | N | N | N |
| **5.3 ... multiple analyses of the data?** | N | N | N | Y | Y | N |
| **Risk-of-bias judgement** | Low | Low | Low | Some concerns | Some concerns | Low |
| Optional: What is the predicted direction of bias due to selection of the reported result? | Low | Low | Low | High | Some concerns | Low |

Overall risk of bias

|  | Abdul Waheb et al | Kooistra et al | Lanz et al | Linke et al | Makkar et al (BEV Vs EV/Portico) | Thiele et al |
| --- | --- | --- | --- | --- | --- | --- |
| **Risk of Bias judgement** | Low | Low | Low | High | Some concern | Low |

**Table S3: Baseline characteristics of included studies**

|  | **Abdel-Wahab 2014** | | **Kooistra 2020** | | **Lanz 2019** | | **Linke 2017** | | **Makkar 2020**  **Makkar 2020 (a)** | | | | **Thiele 2020** | |
| --- | --- | --- | --- | --- | --- | --- | --- | --- | --- | --- | --- | --- | --- | --- |
|  | **BEV** | **SEV** | **BEV** | **SEV** | **BEV** | **SEV** | **BEV** | **SEV** | **BEV** | **SEV -Evolut** | **BEV** | **SEV- Portico** | **BEV** | **SEV** |
| Age ,mean(SD) | 81.9 (6.7) | 79.6 (15.8) | 77 (11) | 81± 7 | 83 (3.9) | 82.6 (4.3) | 81.8(6.7) | 82.9(6.1) | 83.5 (7.4) | 83.3 (6.7) | 83.5 (7.4) | 83 (7.6) | 81.5(5.3) | 81.7 ± 5.3 |
| Women(N,%) | 69(57.0) | 86(71.7) | 15 (52) | 13 (48) | 202 (55%) | 218 (59%) | 255(51) | 127(45) | 107(51.9) | 60(54.1%) | 107(51.9% | 193(51.5%) | 110(50.2) | 114(52.1) |
| Logistic Euro score mean(SD) | 21.5 (12.9) | 22.1 (14.7) | 13 (8) | 16 (10) | - | - | 16.5(10.2) | 17.9(10.5) | - | - | - | - | 14.8(8.6-24.4) | 14.9(8.9-23.8) |
| STS, mean (SD) | 5.6 (2.9) | 6.2 (3.9) | - | - | 3·7% (2·5–4·9) | - | - | - | 6.2 (3.4) | 6.5 (3.1) | 6.2 (3.4) | 6.3 (3.4) | 4.7(3.1-9.4) | - |
| STS prom score median (IQR) | - | - | - | - | 3.4 (2.6 - 5.2) | 3.7 (2.5 - 4.9) | - | - | - | - | - | -- | 4.7 (3.1–9.4) | 4.9 (2.9–9.9) |
| NYHA III OR IV(n/N,%) | 97/121(80.1) | 98/120(81.7) | 16/29 (55) | 13/27 (48) | 268/367 (73) | 287/372 (77) | - | - | 151/206 (73.3) | 61/111 (67.6) | 73.3 | 71.2% | 140/218(68.8) | 141/216(65.2) |
| DM(n/N,%) | 38/121 (31.4) | 32/120 (26.7) | 6/29 (21) | 11/27 (41) | 116/367 (32) | 108/372 (29) | 164(32.8) | 71(25.2) | - | - | - | - | 68/219(31.1) | 79/218(36.2) |
| Hypertension | - | - | 23/29 (79) | 15/27 (56) | 333/367(91) | 341/372(92) | - | - | - | - | - | - | 204/219(93.2) | 193/219 (88.1) |
| HLD | - | - | 18/29 (62) | 18/27 (67) | 211 (57%) 216 (59%) | - | - | -- |  | - | - | 100/218 (45.9) 80/217 (36.9 | 80/217 (36.9 | - |
| CAD(n/N, %) | 73/121 (60.3) | 79/120 (65.8) | 17/29 (59) | 10/27 (37) | 219/367 (60) | 218/372 (59) | 245(49.1) | 151(53.5) | - | - | - | - | 116/219(52.7) | 127/219 (58.0) |
| Prior MI  (n/N, %) | 14/121 (11.6) | 16/120 (13.3) | 7/29 (24) | 7/27 (26) | 47/367 (13) | 39/372 (10) | 64(13) | 48(17.1) | - | - | - | - | 22/219(10) | 19/219(8.7) |
| Prior CABG  (n/N, %) | 19/121 (15.7) | 15/120 (12.5) | 5/29 (17) | 3/27 (11) | 30/367 (8) | 31/372 (8) | 65(13) | 48(17) | - | - | - | - | 18/219(8.2) | 26/219(11.9) |
| Prior PCI (n/N, %) | 44/121 (36.4) | 51/120 (42.5) | 10/29 (35) | 9/27 (33) | 126/367 (34) | 117/372 (31) |  | -- |  | -- | - | - | 79/219(36.1) | 84/219(38.4) |
| Prior Cerebral vascular disease  (n/N, %) | 26/121 (21.5) | 22/120 (18.3) | 5/29 (17) | 4/27 (15) | 47/367 (13) | 47/372 (13) | 48(9.6) | 32(11.3) | - | - | - | - | 26/219(11.9) | 25/219(11.4) |
| Prior PAD (n/N, %) | 20/121 (16.5) | 22/120 (18.3) | 3/29 (10) | 6/27 (22) | 40/367 (11) | 46/372 (12) | 73(14.6) | 42(14.9) | - | - | -- | - | 26/220(11.8) | 29/218(13.3) |
| Pulmonary disease | 27/121 (22.3) | 24/120 (20.0) | 1/29 (3) | 7/27 (26) | 44/367 (12) | 33/372 (9) | 90(18) | 65(23) | - | - | - | - | 29/217(13.4) | 30/219(13.7) |
| Creatinine level, mean (SD) | 1.1 (0.4) | 1.2 (0.5) | - | - | - | - | - | - | - | - | - | - | - | - |
| Severe CKD- GFR(<30 ml/min) | 7/121 (5.8) | 10/120 (8.3) | - | - | - | - | 26(5.2) | 12(4.3) | - |  | -- | - | 184/214 (86.0) |  |
| AF mean (SD) | 39/117 (33.3) | 29/117 (24.8) | 10/29 (34) | 7/27 (26) | 136/367 (37) | 133/372 (36) | 192(38.6) | 94(33.3) | - | - | - | - | 39/219(42.5) | 103/219(47.0) |
| Prior PPM mean (SD) | 7/117(5.9) | 9/117 (7.7) | 2/29 (7) | 0 | 36/367 (10) | 43/372 (12) | - | - | - | - | -- | - | 23/219(10.5) | 24/218(11) |
| LVEF ,mean(SD)% | 52.5 (13.8) | 54.9 (11.9) | 53 (10) | 51 (11) | 57.1 (10.7) | 56.4 (11.1) | 54.2(12.7) | 52.5(12.9) | - | - | - | - | - | - |
| Echo- AVA, mean(SD) | 0.7 (0.2) | 0.7 (0.2) | 0.72 (0.2) | 0.75 (0.3) | 0.7(0.2), N = 364 | 0.7(0.2), N = 368 | - | - | 0.68 (0.16) | 0.67 (0.17) | 0.68 (0.16) | 0.69 (0.18) | 0.8(0.6-0.9) | 0.7(0.6-0.9) |
| Echo- mean aortic gradient  mean(SD) | 43.3 (15.4) | 43.0 (13.9) | 42 (13) | 44 (20) | 41.5(15.1) | 42.9(17.2) | - | - | 46.7 (11.7) | 46.3 (10.8) | 46.7 (11.7) | 46.2 (11.2) | 37(26.5-47.5) | 38.5(30.0-50.5) |
| CT- AV annulus area, (mean, SD) | 456.6 (70.2) | 432.3 (75.3) | - | - | 442.9 (60.3) | 439.1 (59.6) | - | - | - | - | - | - | - | - |
| CT-AV Perimeter,( mean, SD) | 78.1 (5.9) | 75.3 (6.6) | - | - | 75.9 (5.1) | 75.7 (5.2) | - | - | - | - | - | - | - | - |
| CT-AV cusp severe Ca | 33/94 (35.1) | 40/93 (43.0) | - | - | - | - | - | - | - | - | - | - | - | - |
| CT- LVOT severe Ca(n/N,%) | 5/94 (5.3) | 6/93 (6.5) | - | - |  | -- | - | - | - | - | - | - | - | - |
| Euroscore II,median (IQR) | 4.6 (2.9-7.9) | 4.4 (2.4-7.2) | - | - | - | - | - | - | 6.8 (5.9) | 5.2 (4.4) | 6.8 (5.9) | 6.6 (7.2) | 3.8(2.4-6.1) | 4.1 (2.5–7.5) |
| eGFR, mean (SD) | - | - | 65 (20) | 61 (23) | - | - | - | - | - | - | - | - | - | - |
| Creatinine >2% | - | - | - | - | 17/367(5%) | 15/372(4%) | - | - | - | - | -- | - | - | - |
| STS prom score median (IQR) | - | - | - | - | 3.4 (2.6 - 5.2) | 3.7 (2.5 - 4.9) | - | - | - | - | - | - | 4.7 (3.1–9.4) | 4.9 (2.9–9.9) |

— indicates data not available

Baseline characteristics were well-balanced in all studies, except for higher prevalence of female sex in Addul-Waheb et al study.

STS PROM = Society of Thoracic Surgeons Predicted Risk of Mortality; NYHA = New York Heart Association; TIA = transient ischemic attack; CAD = coronary artery disease; PCI = percutaneous coronary intervention; CABG = coronary artery bypass grafting; MI = myocardial infarction; PPM/ICD = permanent pacemaker/implantable cardioverter-defibrillator; AVA = aortic valve area; LVEF = left ventricular ejection fraction; BAV = balloon aortic valvuloplasty; N/A = not applicable.

**Table S4: Sensitivity Analysis Using Fixed-Effect Models**

| **Outcome** | **RR (95% CI)** | **P Value** | **I^2^** |
| --- | --- | --- | --- |
| All-Cause Death | 0.43(0.28-0.67) | <0.0002 | 6 |
| Cardiovascular Death | 0.52(0.31-0.87) | 0.01 | 0 |
| Stroke | 1.43(0.88-2.30) | 0.15 | 51 |
| Implantation of more than one valve per procedure | 0.15(0.07-0.31) | <0.00001 | 0 |
| Moderate/Severe AR/PVL | 0.28(0.17-0.47) | <0.00001 | 0 |
| PPM Implantation | 0.74(0.59-0.94) | 0.01 | 42 |
| Myocardial Infarction | 0.39(0.13-1.13) | 0.08 | 0 |
| Life-Threatening/Disabling Bleeding | 0.84(0.61-1.17) | 0.30 | 24% |
| Major Vascular Complications | 0.80(0.60-1.07) | 0.13 | 0 |
| Major bleeding | 1.26(0.76-2.06) | 0.37 | 0 |
| Rehospitalization for valve related symptoms | 0.56(0.22-1.45) | 0.23 | 35 |
| Valve related dysfunction requiring repeat procedure | 0.40(0.08-2.03) | 0.27 | 0 |
| Valve malposition | 0.33(0.09-1.22) | 0.10 | 0 |
| AKI Stage 2/3 | 0.64(0.40-1.01) | 0.06 | 48% |
| New/Worsening Atrial Fibrillation | 0.75(0.46-1.22) | 0.24 | 30 |
| Early safety | 0.85(0.66-1.09) | 0.19 | 61 |
| Clinical Efficacy | 1.08(0.89-1.31) | 0.42 | 0 |

PPM = permanent pacemaker; AR= Aortic regurgitation AKI = acute kidney injury; PVL = paravalvular leak

**Table.S5. Summary of Findings- GRADE Approach for clinical outcomes**

| **Summary of findings:** | | | | | | |
| --- | --- | --- | --- | --- | --- | --- |
| **Balloon expanding trans-catheter valve platform compared to Self-expanding trans-catheter valve platform for Patients with severe aortic stenosis** | | | | | | |
| **Patient or population**: Patients with severe aortic stenosis  **Setting**: Randomized control trials  **Intervention**: Balloon expanding trans-catheter valve platform  **Comparison**: Self expanding trans-catheter valve platform | | | | | | |
| Outcome № of participants  (studies) | Relative effect (95% CI) | **Anticipated absolute effects (95% CI)** | | | Certainty | What happens |
|  |  |  |  | **Difference** |  |  |
| Device success as per study protocol - Device success as defined by VARC № of participants: 1396 (4 RCTs) | **RR 0.99** (0.77 to 1.28) | 76.3% | **75.5%** (58.7 to 97.6) | **0.8% fewer** (17.5 fewer to 21.4 more) | ⨁⨁◯◯ LOW ^a,b^ |  |
| All cause mortality № of participants: 2935 (6 RCTs) | **RR 0.51** (0.31 to 0.82) | 4.5% | **2.3%** (1.4 to 3.7) | **2.2% fewer** (3.1 fewer to 0.8 fewer) | ⨁⨁⨁⨁ HIGH ^c^ |  |
| All cause mortality - 30 days( Confining only to RCT and post hoc results) № of participants: 2153 (5 RCTs) | **RR 0.44** (0.20 to 1.00) | 3.9% | **1.7%** (0.8 to 3.9) | **2.2% fewer** (3.1 fewer to 0 fewer) | ⨁⨁⨁⨁ HIGH ^d^ |  |
| All cause mortality - 30 days mortality difference BEV Vs RR-SEV № of participants: 1128 (2 RCTs) | **RR 0.24** (0.01 to 4.72) | 4.1% | **1.0%** (0 to 19.5) | **3.1% fewer** (4.1 fewer to 15.3 more) | ⨁⨁⨁◯ MODERATE ^c,d,e^ |  |
| All stroke (disabling and non-disabling) № of participants: 2231 (5 RCTs) | **RR 1.43** (0.88 to 2.30) | 2.4% | **3.4%** (2.1 to 5.5) | **1.0% more** (0.3 fewer to 3.1 more) | ⨁◯◯◯ VERY LOW ^c,f,g^ |  |
| Cardiovascular mortality - 30 days № of participants: 1807 (4 RCTs) | **RR 0.54** (0.32 to 0.90) | **Study population** | | | ⨁◯◯◯ VERY LOW ^h,i^ |  |
|  |  | 4.3% | **2.3%** (1.4 to 3.9) | **2.0% fewer** (2.9 fewer to 0.4 fewer) |  |  |
|  |  | **Moderate** | | |  |  |
|  |  | 4.3% | **2.3%** (1.4 to 3.8) | **2.0% fewer** (2.9 fewer to 0.4 fewer) |  |  |
| Life-threatening bleeding - 30 days № of participants: 2244 (5 RCTs) | **RR 0.84** (0.54 to 1.30) | 6.2% | **5.2%** (3.4 to 8.1) | **1.0% fewer** (2.9 fewer to 1.9 more) | ⨁⨁◯◯ LOW ^g,h^ |  |
| Acute kidney injury (including renal replacement therapy) № of participants: 1453 (4 RCTs) | **RR 0.57** (0.27 to 1.19) | 5.8% | **3.3%** (1.6 to 6.9) | **2.5% fewer** (4.2 fewer to 1.1 more) | ⨁⨁◯◯ LOW ^g^ |  |
| Major vascular complication - 30 days № of participants: 2244 (5 RCTs) | **RR 0.81** (0.61 to 1.08) | 8.5% | **6.9%** (5.2 to 9.2) | **1.6% fewer** (3.3 fewer to 0.7 more) | ⨁⨁◯◯ LOW ^g,h,j^ |  |
| Valve-related dysfunction requiring repeat procedure (BAV, TAVI, or SAVR) - 30 days № of participants: 1025 (3 RCTs) | **RR 0.40** (0.08 to 2.06) | 1.0% | **0.4%** (0.1 to 2) | **0.6% fewer** (0.9 fewer to 1 more) | ⨁⨁⨁⨁ HIGH ^g,j^ |  |
| Implant >1 valve - Day 0 № of participants: 1818 (4 RCTs) | **RR 0.56** (0.13 to 2.44) | 5.1% | **2.9%** (0.7 to 12.5) | **2.3% fewer** (4.5 fewer to 7.4 more) | ⨁⨁⨁◯ MODERATE ^g,h^ |  |
| Requiring hospitalizations for valve-related symptoms or worsening congestive heart failure - 30 days № of participants: 1023 (3 RCTs) | **RR 0.99** (0.32 to 3.11) | 2.2% | **2.1%** (0.7 to 6.7) | **0.0% fewer** (1.5 fewer to 4.5 more) | ⨁⨁◯◯ LOW ^g,k^ |  |
| New PPM requirement - 30 days № of participants: 1425 (4 RCTs) | **RR 0.73** (0.52 to 1.02) | 18.4% | **13.4%** (9.5 to 18.7) | **5.0% fewer** (8.8 fewer to 0.4 more) | ⨁◯◯◯ VERY LOW ^g,l^ |  |
| Valve malposition - day 0 № of participants: 1036 (3 RCTs) | **RR 0.34** (0.09 to 1.22) | 1.5% | **0.5%** (0.1 to 1.9) | **1.0% fewer** (1.4 fewer to 0.3 more) | ⨁⨁◯◯ LOW ^g,h^ |  |
| Moderate and severe AR/Paravalvular AR - 30 days № of participants: 1424 (4 RCTs) | **RR 0.29** (0.17 to 0.48) | 9.0% | **2.6%** (1.5 to 4.3) | **6.4% fewer** (7.5 fewer to 4.7 fewer) | ⨁⨁⨁⨁ HIGH ^g^ |  |
| Major bleeding - 30 days № of participants: 730 (3 RCTs) | **RR 1.26** (0.77 to 2.08) | 6.6% | **8.4%** (5.1 to 13.8) | **1.7% more** (1.5 fewer to 7.2 more) | ⨁⨁⨁◯ MODERATE ^g^ |  |
| Myocardial infarction - 30 days № of participants: 1807 (4 RCTs) | **RR 0.37** (0.11 to 1.18) | 1.0% | **0.4%** (0.1 to 1.2) | **0.6% fewer** (0.9 fewer to 0.2 more) | ⨁⨁◯◯ LOW ^g^ |  |
| Atrial fibrillation - 30 days № of participants: 1513 (2 RCTs) | **RR 0.76** (0.42 to 1.38) | 4.6% | **3.5%** (1.9 to 6.4) | **1.1% fewer** (2.7 fewer to 1.8 more) | ⨁◯◯◯ VERY LOW ^c,g^ |  |
| Early safety - 30 days № of participants: 1463 (4 RCTs) | **RR 0.86** (0.54 to 1.37) | 15.9% | **13.7%** (8.6 to 21.8) | **2.2% fewer** (7.3 fewer to 5.9 more) | ⨁⨁⨁◯ MODERATE ^g^ |  |
| Clinical Efficacy - 30 days № of participants: 1124 (2 RCTs) | **RR 1.09** (0.89 to 1.32) | 25.3% | **27.5%** (22.5 to 33.4) | **2.3% more** (2.8 fewer to 8.1 more) | ⨁⨁⨁◯ MODERATE ^g^ |  |
| ***The risk in the intervention group** (and its 95% confidence interval) is based on the assumed risk in the comparison group and the **relative effect** of the intervention (and its 95% CI).   **CI:** Confidence interval; **RR:** Risk ratio | | | | | | |
| **GRADE Working Group grades of evidence** **High certainty:** We are very confident that the true effect lies close to that of the estimate of the effect **Moderate certainty:** We are moderately confident in the effect estimate: The true effect is likely to be close to the estimate of the effect, but there is a possibility that it is substantially different **Low certainty:** Our confidence in the effect estimate is limited: The true effect may be substantially different from the estimate of the effect **Very low certainty:** We have very little confidence in the effect estimate: The true effect is likely to be substantially different from the estimate of effect | | | | | | |

**Footnotes**^1^ I^2^-95%, ^2^ Upper limit of CI is >1.25, ^3^ Linke et al with weight of the study being 42.5% was randomized for drug and the study effect was stratified by device or valve randomization laterMakkar et al - Both the study was post hoc analysis, ^4^ Total deaths are less than 300- 400, ^5^ Makkar et al- studies are post hoc analysis,^6^ Makkar et al- studies are post hoc analysis; But their weight is minimal on the analysis,^7^ I^2^-61%,^8^ I^2^- 39 %,^9^ Total events less than 300; CI goes beyond < 0.75->1.25,^10^ I^2^>25%,^11^ Linke et al with weight of the study being 42.5% was randomized for drug and the study effect was stratified by device or valve randomization later; Makkar et al - was post hoc analysis,^12^ Makkar et al study is a post hoc analysis,^13^ Makkar et al study is a post hoc analysis; But weight of that study in analysis is only 4%; Its absence does not affect the result significantly,^14^ PORTICO-IDE Trial presented by Makkar et al and Thiele et al did not reveal the 30 day outcomes, though they have mentioned all-cause mortality,^15^ Linke et al with weight of the study being 42.5% was randomized for drug and the study effect was stratified by device or valve randomization later,^16^ I2>25%,^17^ I2-66%,^18^ Makkar et al study is a post hoc analysis; But wt of that study in analysis is only 10%; Its absence does not affect the result significantly,^19^ PORTICO-IDE Trial presented by makkar et al did not reveal the 30 day outcomes, though they have mentioned all-cause mortality,^20^ Abdul Waheb et al, Kooistra et al, and Thiele et al did not report AF outcomes

**TABLE S6 : GRADE Evidence Profile**

**Bibliography**:

1. Abdel-Wahab M, Mehilli J, Frerker C, Neumann F-J, Kurz T, Tölg R, et al. Comparison of balloon-expandable vs self-expandable valves in patients undergoing transcatheter aortic valve replacement: the CHOICE randomized clinical trial. JAMA. 2014 Apr 16;311(15):1503–14
2. Kooistra NHM, Abawi M, Voskuil M, Urgel K, Samim M, Nijhoff F, et al. Randomised comparison of a balloon-expandable and self-expandable valve with quantitative assessment of aortic regurgitation using magnetic resonance imaging. Neth Heart J Mon J Neth Soc Cardiol Neth Heart Found. 2020 Apr 3
3. Lanz J, Kim W-K, Walther T, Burgdorf C, Möllmann H, Linke A, et al. Safety and efficacy of a self-expanding versus a balloon-expandable bioprosthesis for transcatheter aortic valve replacement in patients with symptomatic severe aortic stenosis: a randomised non-inferiority trial. Lancet Lond Engl. 2019 02;394(10209):1619–28.
4. Linke A, Chandrasekhar J, Sartori S, Lefevre T, van Belle E, Schaefer U, et al. Effect of valve design and anticoagulation strategy on 30-day clinical outcomes in transcatheter aortic valve replacement: Results from the BRAVO 3 randomized trial. Catheter Cardiovasc Interv Off J Soc Card Angiogr Interv. 2017 Nov 15;90(6):1016–26.
5. Maisano PF. PORTICO: A Randomized Trial of Portico vs. Commercially Available Transcatheter Aortic Valves in Patients With Severe Aortic Stenosis [Internet]. TCTMD.com. [cited 2020 Apr 29]. Available from: https://www.tctmd.com/slide/portico-randomized-trial-portico-vs-commercially-available-transcatheter-aortic-valves
6. Thiele H, Kurz T, Feistritzer H-J, Stachel G, Hartung P, Eitel I, et al. Comparison of newer generation self-expandable vs. balloon-expandable valves in transcatheter aortic valve implantation: the randomized SOLVE-TAVI trial. Eur Heart J. 2020 Feb 12;

| **Balloon expanding trans-catheter valve platform compared to Self expanding trans-catheter valve platform for Patients with severe aortic stenosis** | | | | | | | | | | | |
| --- | --- | --- | --- | --- | --- | --- | --- | --- | --- | --- | --- |
| **Certainty assessment** | | | | | | | **Summary of findings** | | | | |
| **Participants  (studies) Follow up** | **Risk of bias** | **Inconsistency** | **Indirectness** | **Imprecision** | **Publication bias** | **Overall certainty of evidence** | **Study event rates (%)** | | **Relative effect (95% CI)** | **Anticipated absolute effects** | |
|  |  |  |  |  |  |  | **With Self expanding trans-catheter valve platform** | **With Balloon expanding trans-catheter valve platform** |  | **Risk with Self expanding trans-catheter valve platform** | **Risk difference with Balloon expanding trans-catheter valve platform** |
| **Device success as per study protocol - Device success as defined by VARC** | | | | | | | | | | | |
| 1396 (4 RCTs) | not serious | very serious ^a^ | not serious | serious ^b^ | dose response gradient | ⨁⨁◯◯ LOW | 530/695 (76.3%) | 482/701 (68.8%) | **RR 0.99** (0.77 to 1.28) | 763 per 1,000 | **8 fewer per 1,000** (from 175 fewer to 214 more) |
| **All cause mortality** | | | | | | | | | | | |
| 2935 (6 RCTs) | serious ^c^ | not serious | not serious | not serious | strong association | ⨁⨁⨁⨁ HIGH | 67/1496 (4.5%) | 32/1439 (2.2%) | **RR 0.51** (0.31 to 0.82) | 45 per 1,000 | **22 fewer per 1,000** (from 31 fewer to 8 fewer) |
| **All cause mortality - 30 days( Confining only to RCT and post hoc results)** | | | | | | | | | | | |
| 2153 (5 RCTs) | serious ^d^ | not serious | not serious | not serious | strong association all plausible residual confounding would suggest spurious effect, while no effect was observed | ⨁⨁⨁⨁ HIGH | 47/1214 (3.9%) | 13/939 (1.4%) | **RR 0.44** (0.20 to 1.00) | 39 per 1,000 | **22 fewer per 1,000** (from 31 fewer to 0 fewer) |
| **All cause mortality - 30 days mortality difference BEV Vs RR-SEV** | | | | | | | | | | | |
| 1128 (2 RCTs) | very serious ^c,d^ | serious ^e^ | not serious | serious | very strong association all plausible residual confounding would suggest spurious effect, while no effect was observed | ⨁⨁⨁◯ MODERATE | 29/703 (4.1%) | 5/425 (1.2%) | **RR 0.24** (0.01 to 4.72) | 41 per 1,000 | **31 fewer per 1,000** (from 41 fewer to 153 more) |
| **All stroke (disabling and non-disabling)** | | | | | | | | | | | |
| 2231 (5 RCTs) | serious ^c^ | serious ^f^ | not serious | serious ^g^ | none | ⨁◯◯◯ VERY LOW | 24/1003 (2.4%) | 43/1228 (3.5%) | **RR 1.43** (0.88 to 2.30) | 24 per 1,000 | **10 more per 1,000** (from 3 fewer to 31 more) |
| **Cardiovascular mortality - 30 days** | | | | | | | | | | | |
| 1807 (4 RCTs) | serious ^h^ | not serious | not serious | serious | publication bias strongly suspected ^i^ | ⨁◯◯◯ VERY LOW | 34/793 (4.3%) | 25/1014 (2.5%) | **RR 0.54** (0.32 to 0.90) | **Study population** | |
|  |  |  |  |  |  |  |  |  |  | 43 per 1,000 | **20 fewer per 1,000** (from 29 fewer to 4 fewer) |
|  |  |  |  |  |  |  |  |  |  | **Moderate** | |
|  |  |  |  |  |  |  |  |  |  | 43 per 1,000 | **20 fewer per 1,000** (from 29 fewer to 4 fewer) |
| **Life-threatening bleeding - 30 days** | | | | | | | | | | | |
| 2244 (5 RCTs) | serious ^h^ | not serious | not serious | serious ^g^ | none | ⨁⨁◯◯ LOW | 63/1011 (6.2%) | 73/1233 (5.9%) | **RR 0.84** (0.54 to 1.30) | 62 per 1,000 | **10 fewer per 1,000** (from 29 fewer to 19 more) |
| **Acute kidney injury (including renal replacement therapy)** | | | | | | | | | | | |
| 1453 (4 RCTs) | not serious | serious | not serious | serious ^g^ | none | ⨁⨁◯◯ LOW | 42/724 (5.8%) | 27/729 (3.7%) | **RR 0.57** (0.27 to 1.19) | 58 per 1,000 | **25 fewer per 1,000** (from 42 fewer to 11 more) |
| **Major vascular complication - 30 days** | | | | | | | | | | | |
| 2244 (5 RCTs) | serious ^h^ | not serious | not serious | serious ^g^ | none ^j^ | ⨁⨁◯◯ LOW | 86/1011 (8.5%) | 88/1233 (7.1%) | **RR 0.81** (0.61 to 1.08) | 85 per 1,000 | **16 fewer per 1,000** (from 33 fewer to 7 more) |
| **Valve-related dysfunction requiring repeat procedure (BAV, TAVI, or SAVR) - 30 days** | | | | | | | | | | | |
| 1025 (3 RCTs) | not serious | not serious | not serious | serious ^g^ | strong association ^j^ | ⨁⨁⨁⨁ HIGH | 5/511 (1.0%) | 2/514 (0.4%) | **RR 0.40** (0.08 to 2.06) | 10 per 1,000 | **6 fewer per 1,000** (from 9 fewer to 10 more) |
| **Implant >1 valve - Day 0** | | | | | | | | | | | |
| 1818 (4 RCTs) | serious ^h^ | not serious | not serious | serious ^g^ | strong association | ⨁⨁⨁◯ MODERATE | 41/801 (5.1%) | 8/1017 (0.8%) | **RR 0.56** (0.13 to 2.44) | 51 per 1,000 | **23 fewer per 1,000** (from 45 fewer to 74 more) |
| **Requiring hospitalizations for valve-related symptoms or worsening congestive heart failure - 30 days** | | | | | | | | | | | |
| 1023 (3 RCTs) | not serious | serious ^k^ | not serious | serious ^g^ | none | ⨁⨁◯◯ LOW | 11/511 (2.2%) | 6/512 (1.2%) | **RR 0.99** (0.32 to 3.11) | 22 per 1,000 | **0 fewer per 1,000** (from 15 fewer to 45 more) |
| **New PPM requirement - 30 days** | | | | | | | | | | | |
| 1425 (4 RCTs) | serious | serious ^l^ | not serious | serious ^g^ | none | ⨁◯◯◯ VERY LOW | 130/708 (18.4%) | 99/717 (13.8%) | **RR 0.73** (0.52 to 1.02) | 184 per 1,000 | **50 fewer per 1,000** (from 88 fewer to 4 more) |
| **Valve malposition - day 0** | | | | | | | | | | | |
| 1036 (3 RCTs) | serious ^h^ | not serious | not serious | serious ^g^ | none | ⨁⨁◯◯ LOW | 8/519 (1.5%) | 2/517 (0.4%) | **RR 0.34** (0.09 to 1.22) | 15 per 1,000 | **10 fewer per 1,000** (from 14 fewer to 3 more) |
| **Moderate and severe AR/Paravalvular AR - 30 days** | | | | | | | | | | | |
| 1424 (4 RCTs) | not serious | not serious | not serious | serious ^g^ | strong association | ⨁⨁⨁⨁ HIGH | 64/710 (9.0%) | 18/714 (2.5%) | **RR 0.29** (0.17 to 0.48) | 90 per 1,000 | **64 fewer per 1,000** (from 75 fewer to 47 fewer) |
| **Major bleeding - 30 days** | | | | | | | | | | | |
| 730 (3 RCTs) | not serious | not serious | not serious | serious ^g^ | none | ⨁⨁⨁◯ MODERATE | 24/362 (6.6%) | 31/368 (8.4%) | **RR 1.26** (0.77 to 2.08) | 66 per 1,000 | **17 more per 1,000** (from 15 fewer to 72 more) |
| **Myocardial infarction - 30 days** | | | | | | | | | | | |
| 1807 (4 RCTs) | very serious | not serious | not serious | serious ^g^ | strong association | ⨁⨁◯◯ LOW | 8/793 (1.0%) | 4/1014 (0.4%) | **RR 0.37** (0.11 to 1.18) | 10 per 1,000 | **6 fewer per 1,000** (from 9 fewer to 2 more) |
| **Atrial fibrillation - 30 days** | | | | | | | | | | | |
| 1513 (2 RCTs) | very serious ^c^ | not serious | not serious | serious ^g^ | none | ⨁◯◯◯ VERY LOW | 30/649 (4.6%) | 32/864 (3.7%) | **RR 0.76** (0.42 to 1.38) | 46 per 1,000 | **11 fewer per 1,000** (from 27 fewer to 18 more) |
| **Early safety - 30 days** | | | | | | | | | | | |
| 1463 (4 RCTs) | not serious | not serious | not serious | serious ^g^ | none | ⨁⨁⨁◯ MODERATE | 116/730 (15.9%) | 99/733 (13.5%) | **RR 0.86** (0.54 to 1.37) | 159 per 1,000 | **22 fewer per 1,000** (from 73 fewer to 59 more) |
| **Clinical Efficacy - 30 days** | | | | | | | | | | | |
| 1124 (2 RCTs) | not serious | not serious | not serious | serious ^g^ | none | ⨁⨁⨁◯ MODERATE | 142/562 (25.3%) | 154/562 (27.4%) | **RR 1.09** (0.89 to 1.32) | 253 per 1,000 | **23 more per 1,000** (from 28 fewer to 81 more) |

**CI:** Confidence interval; **RR:** Risk ratio

#### Explanations

a. i2-94%

b. Upper limit of CI is >1.24

c. Linke et al with weight of the study being 42.5% was randomized for drug, and the study effect was stratified by device or valve randomization later. Makkar et al - the study was post hoc analysis where they have mentioned all cause mortality only for possible sub-group analysis

d. Makkar et al is a post hoc analysis

e. i2-61%

f. i2- 51 %

g. Total events less than 300; CI goes beyond < 0.75->1.25

h. Linke et al with weight of the study being 42.5% was randomized for drug, and the study effect was stratified by device or valve randomization later

i. Assymmetric funnel plot

j. PORTICO-IDE Trial presented by makkar et al and thiele et al did not reveal the 30 day outcomes, though they have mentioned all cause mortality

k. i2-35%

l. i2-42%

2.2 **Supplementary Figures**

**
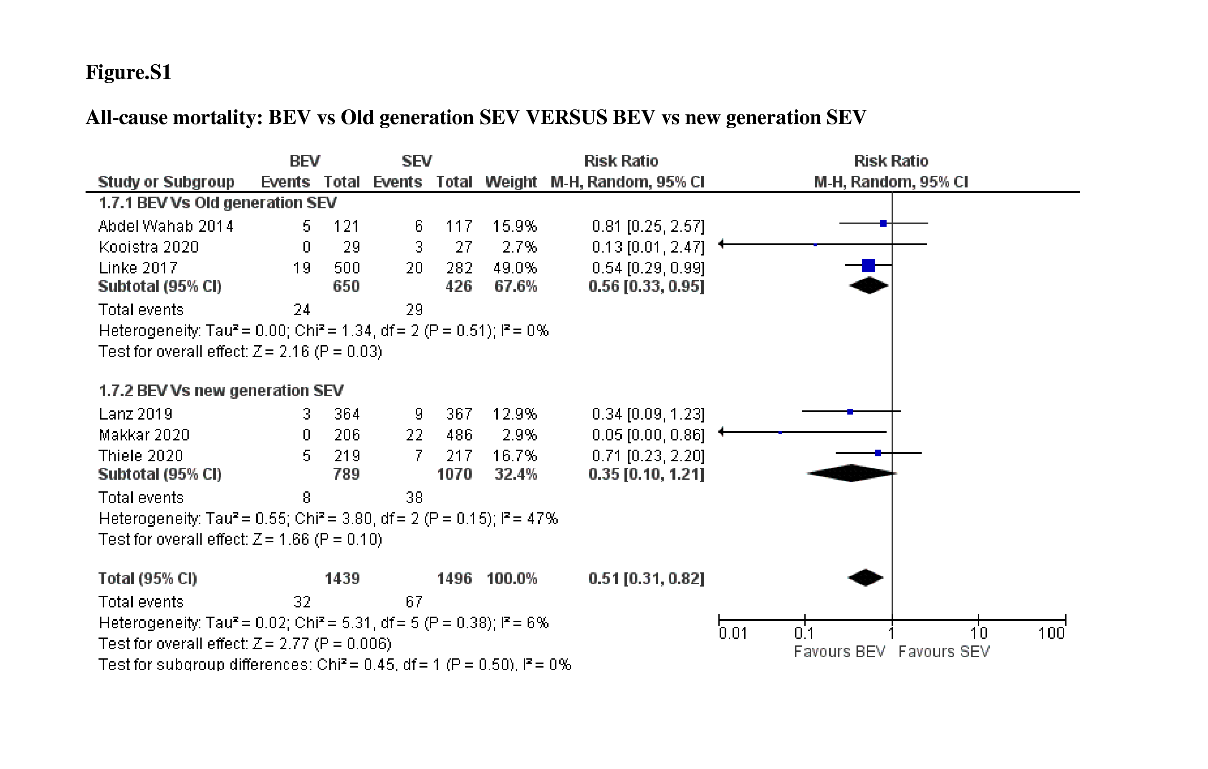
**

**Supplementary Figure 1.** All-cause mortality: BEV vs Old generation SEV VERSUS BEV vs new generation SEV

**
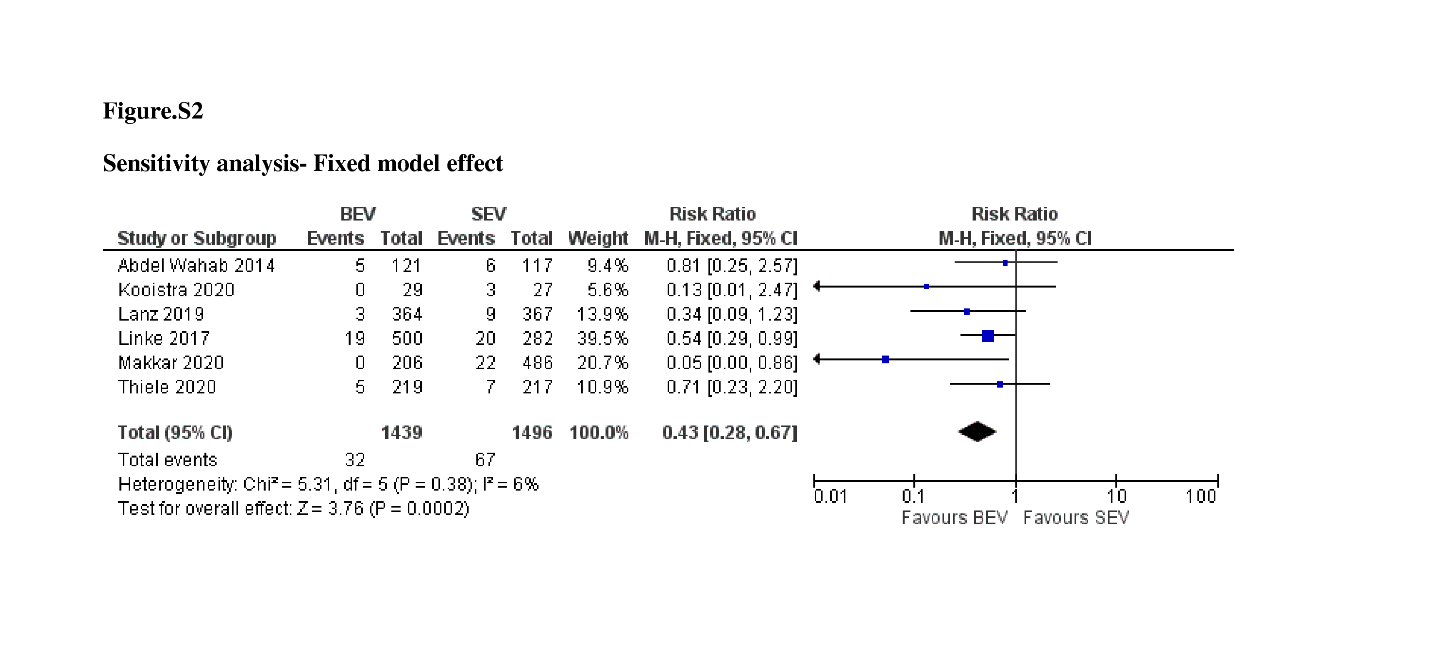
**

**Supplementary Figure 2.** Sensitivity analysis- Fixed model effect


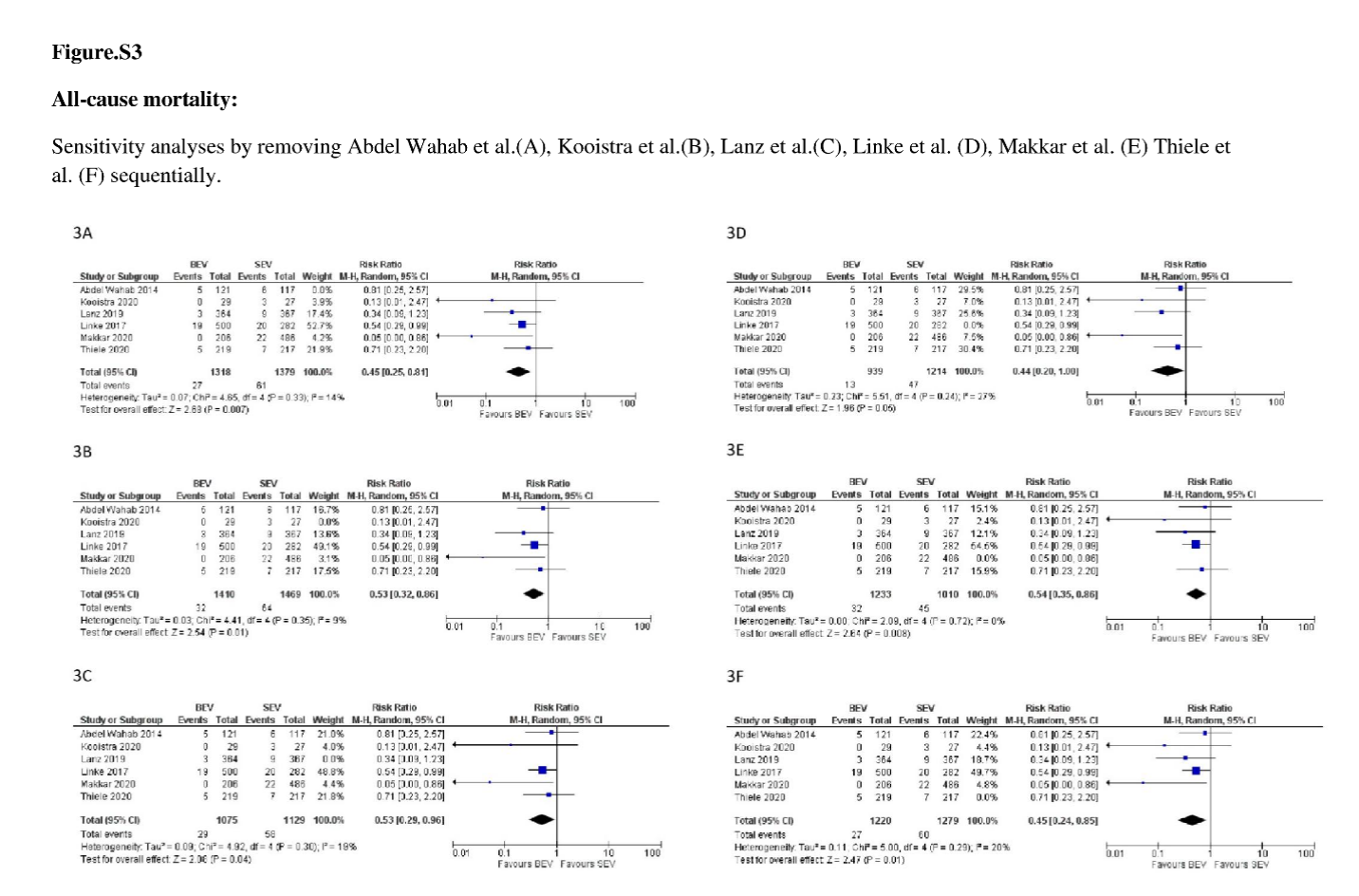


**Supplementary Figure 3.** All cause mortality


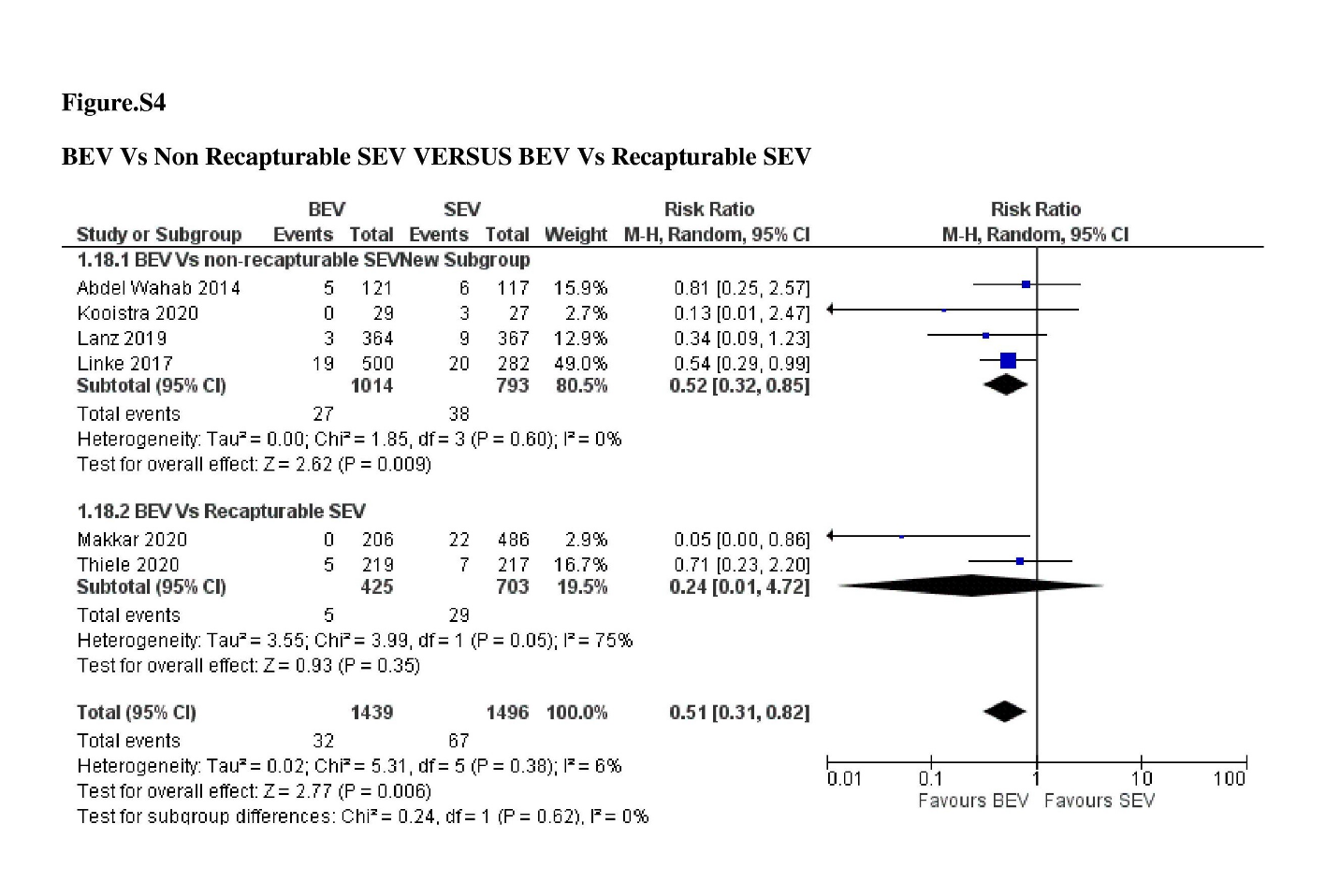


**Supplementary Figure 4.** BEV Vs Non Recapturable SEV VERSUS BEV Vs Recapturable SEV


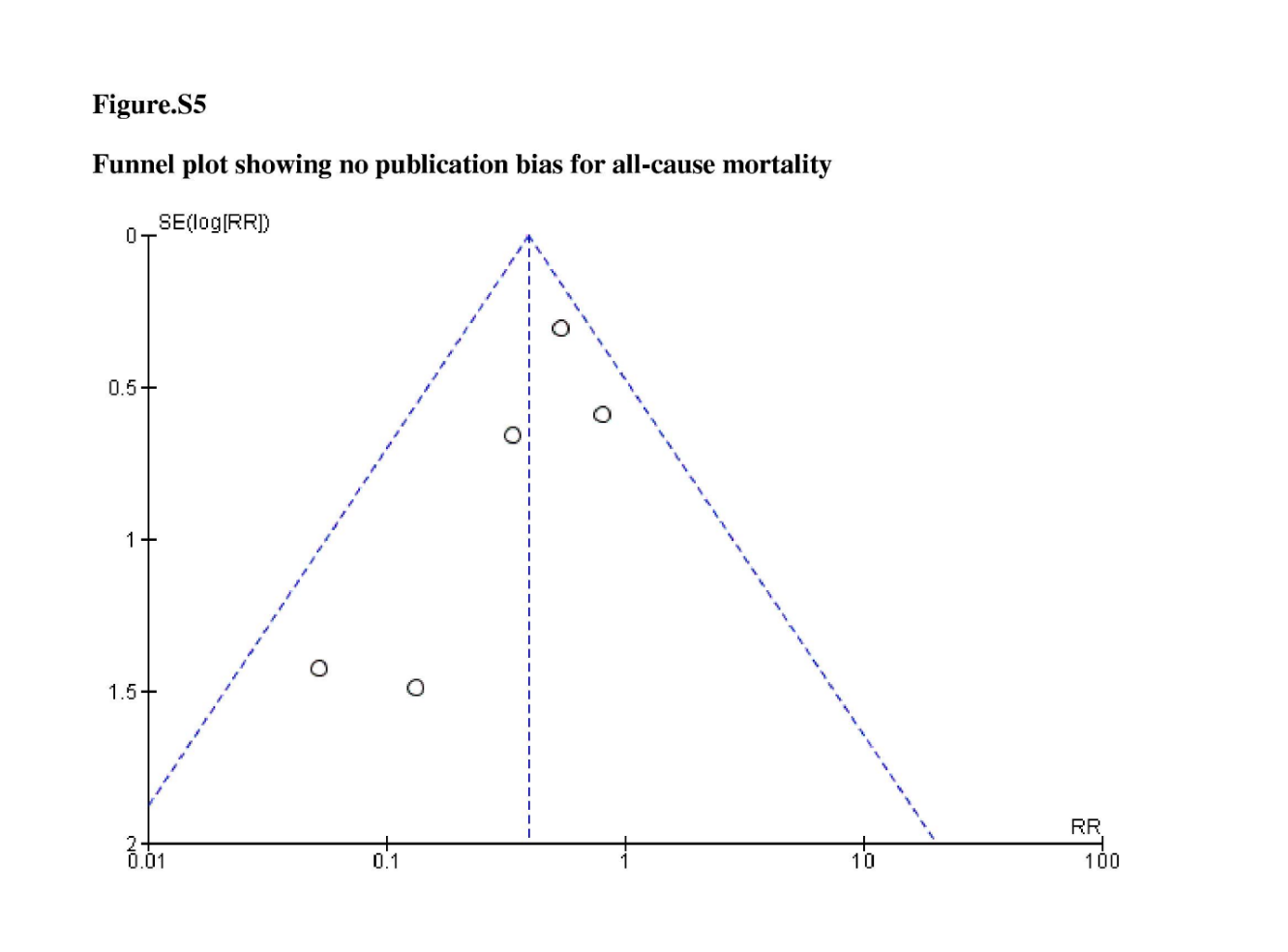


**Supplementary Figure 5.** Funnel plot showing no publication bias for all-cause mortality

**
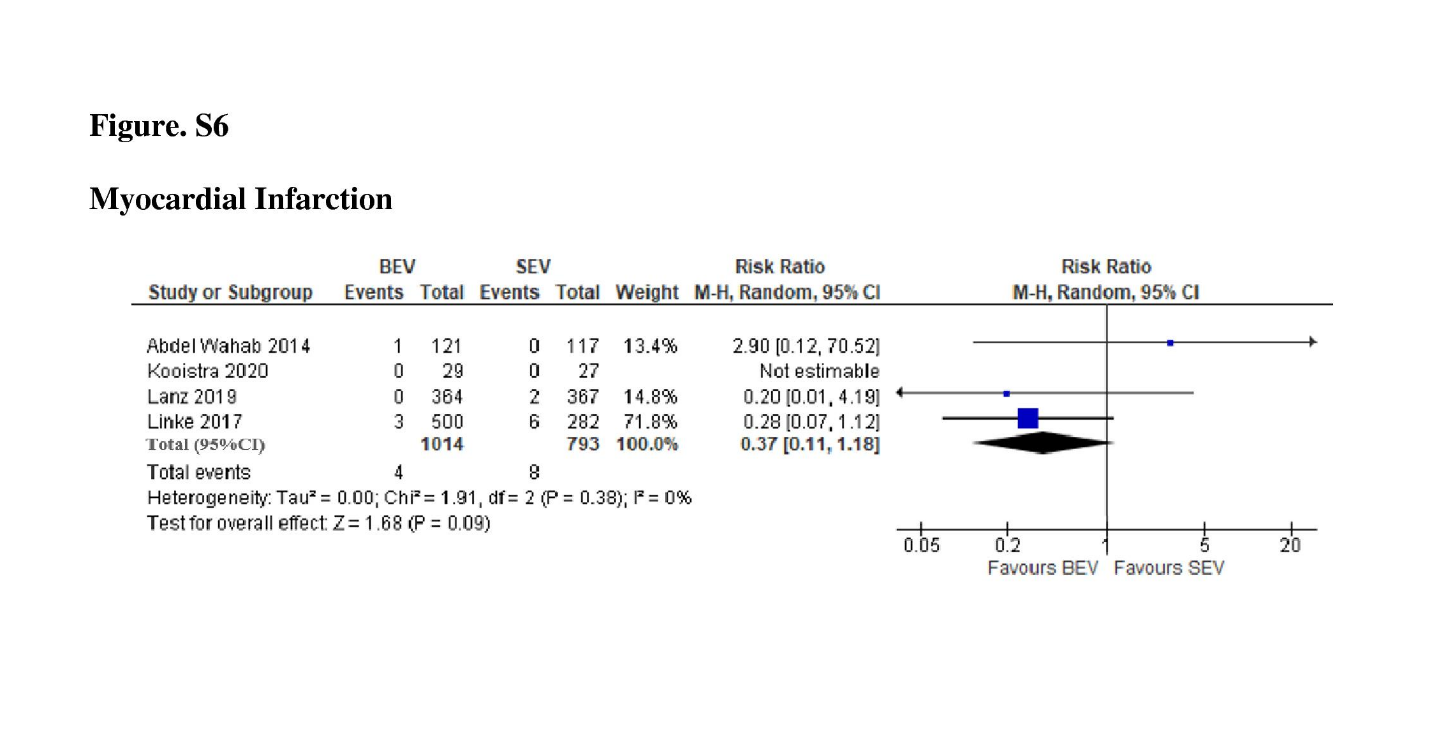
**

**Supplementary Figure 6.** Myocardial Infarction

**
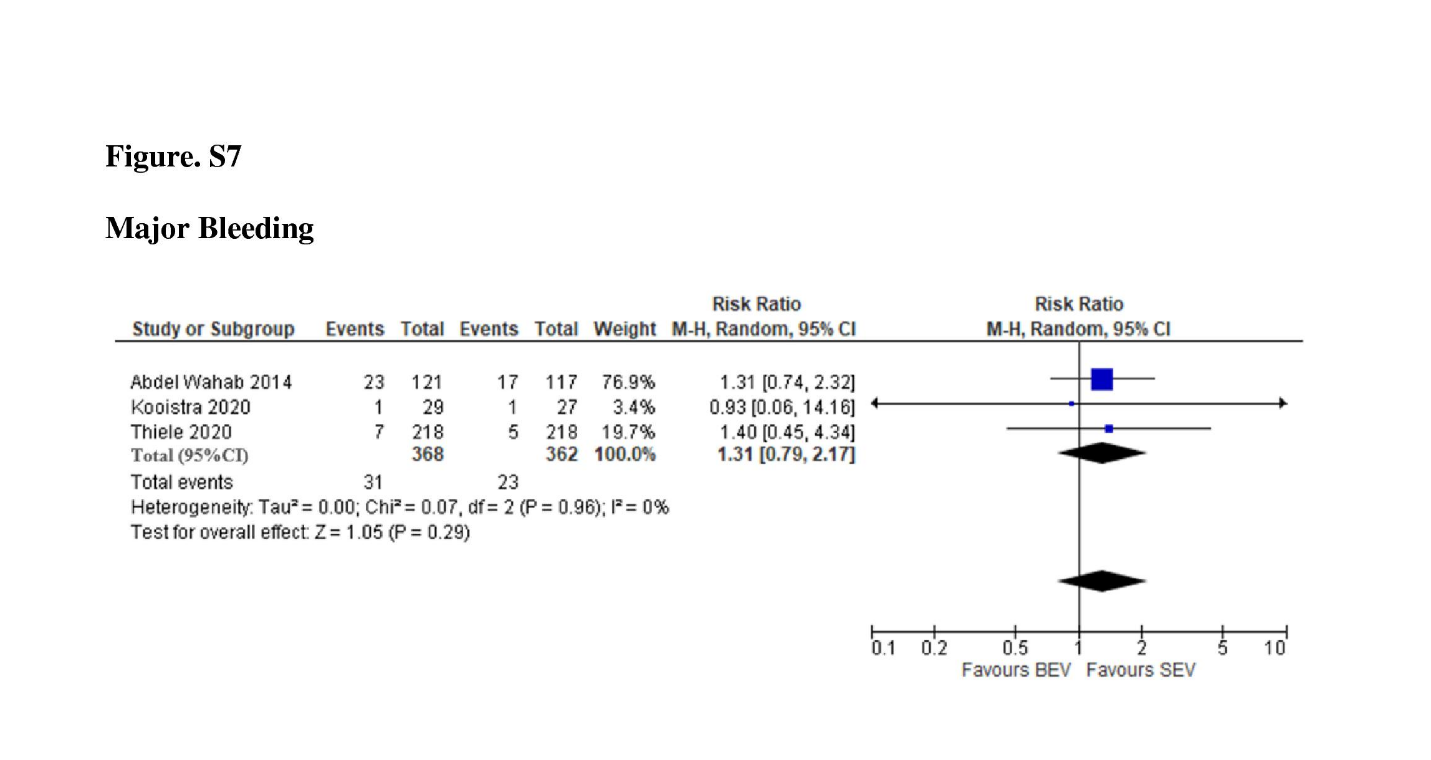
**

**Supplementary Figure 7.** Major Bleeding

**
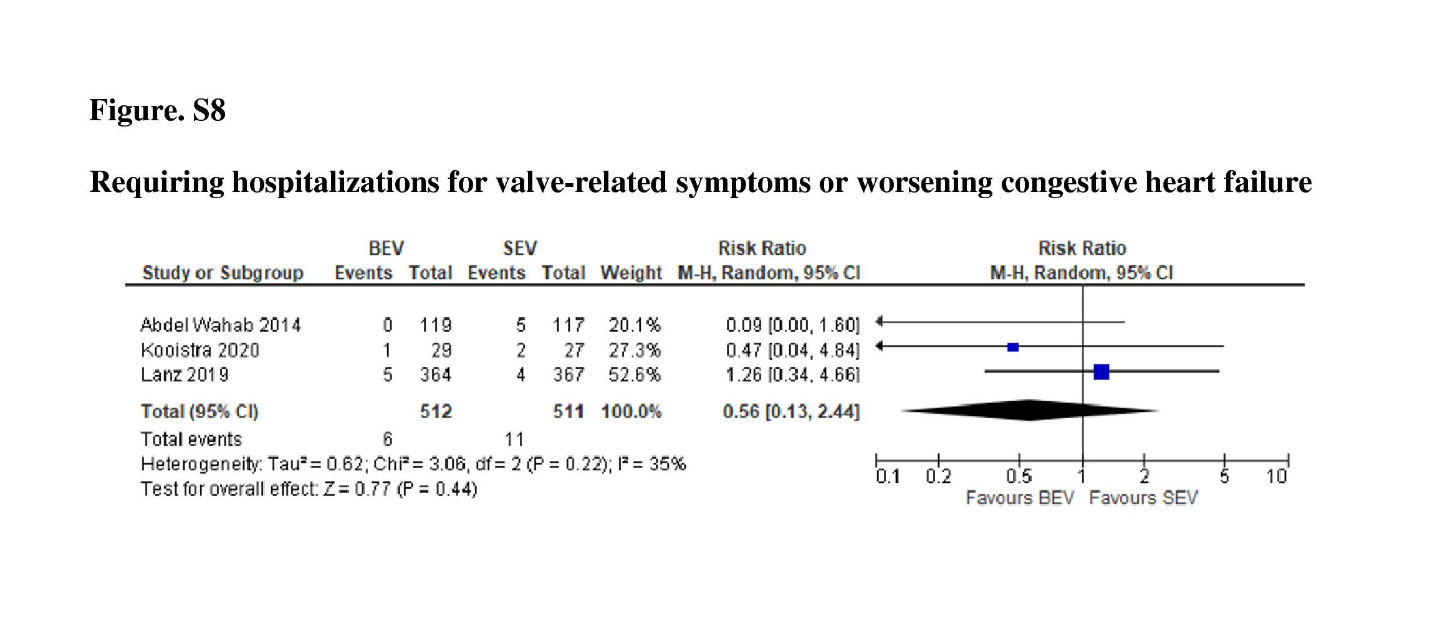
**

**Supplementary Figure 8.** Requiring hospitalizations for valve-related symptoms or worsening congestive heart failure

**
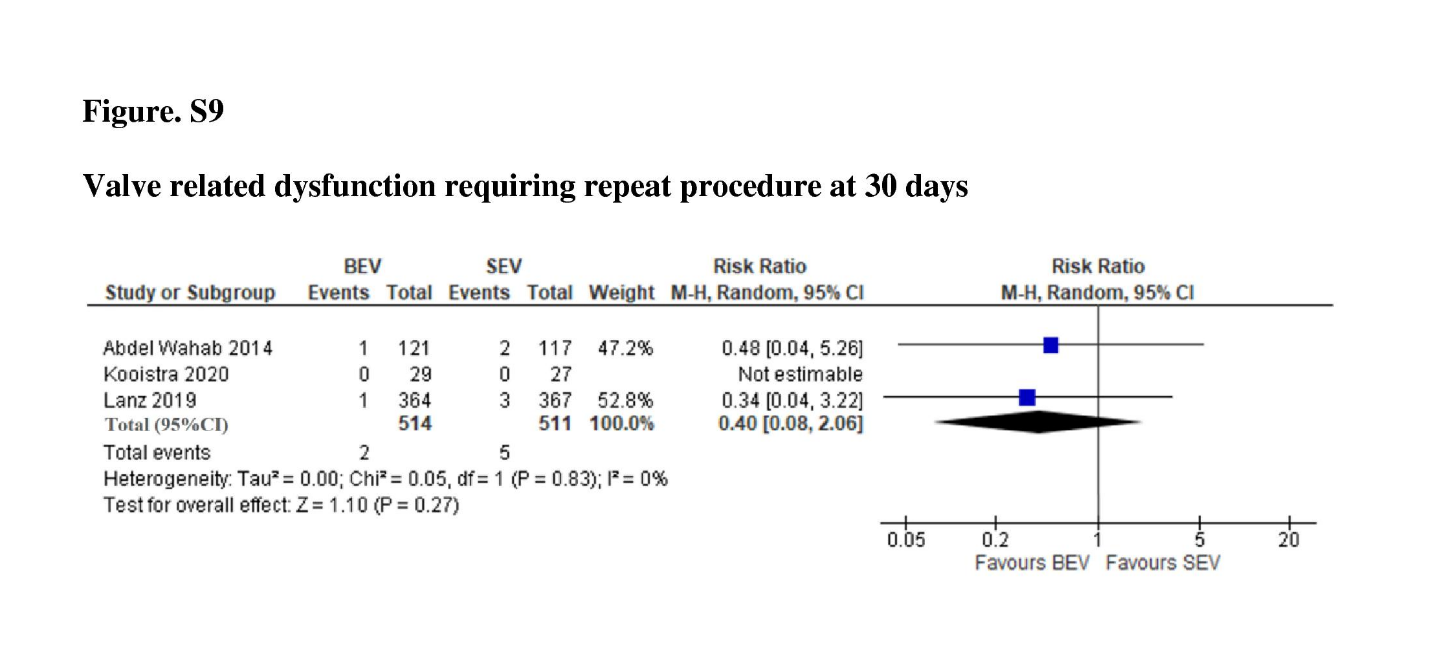
**

**Supplementary Figure 9.** Requiring hospitalizations for valve-related symptoms or worsening congestive heart failure

**
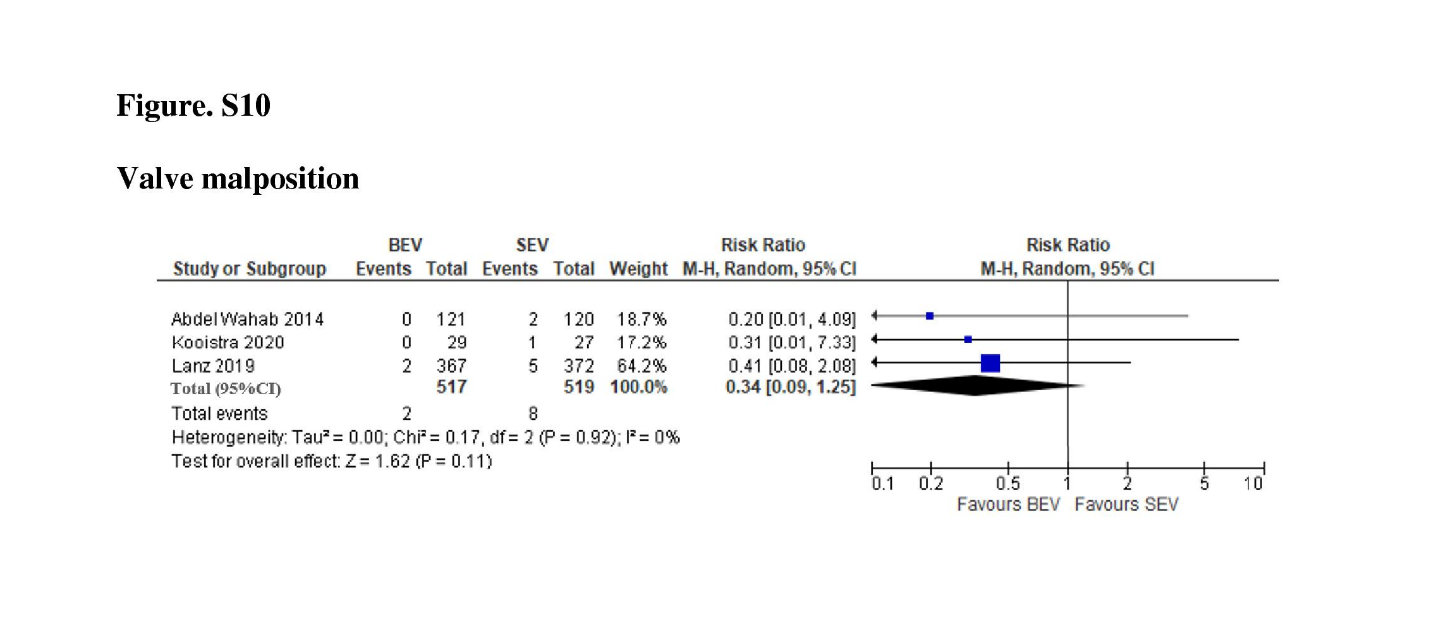
**

**Supplementary Figure 10.** Valve malposition

**
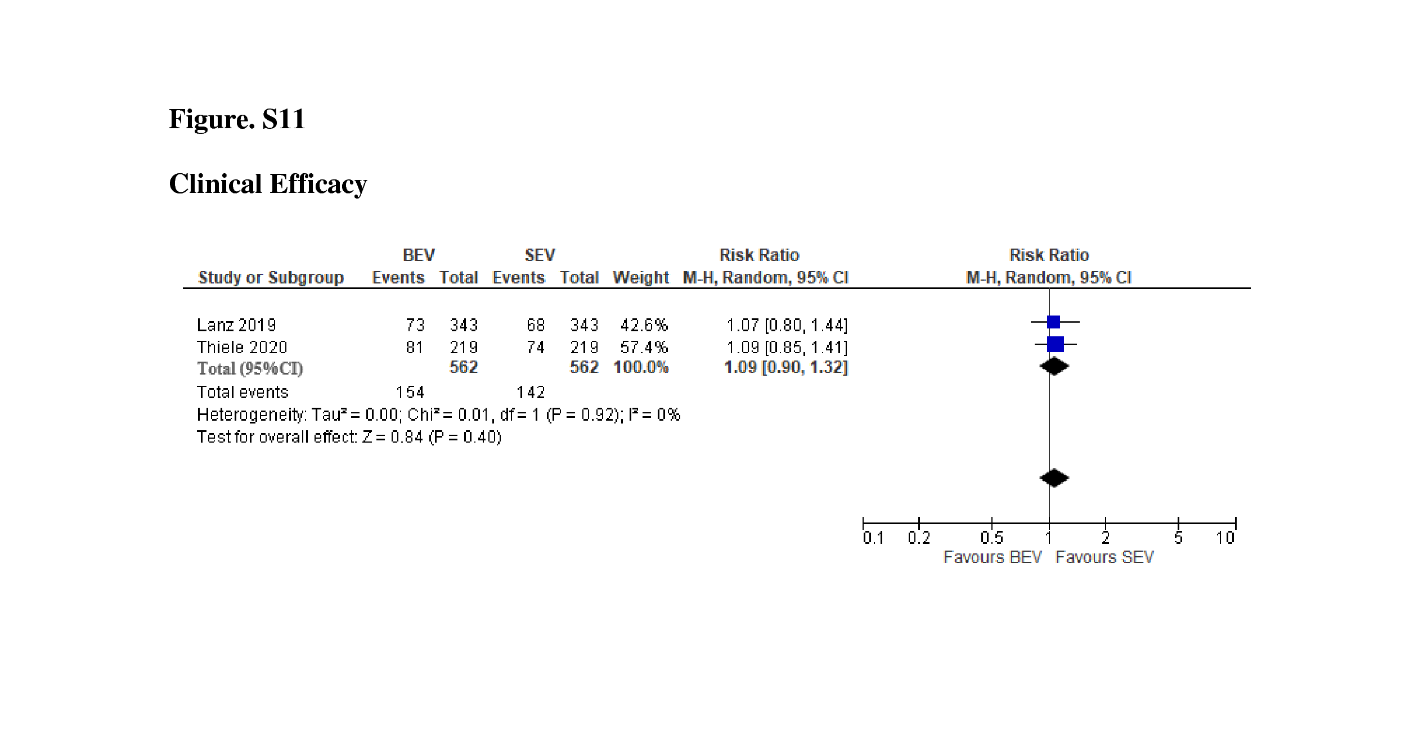
**

**Supplementary Figure 11.** Clinical Efficacy
